# The TaDof-2D–miR1832–TaP450-7A module regulates low-temperature-induced seed dormancy release in wheat

**DOI:** 10.1016/j.xplc.2026.101749

**Published:** 2026-01-30

**Authors:** Wei Gao, Zi-heng Cui, Hua Xie, Jia-jia Cao, Li-tian Zhang, Yu-xia Lv, Bing-bing Tian, Chao-xu He, Zi-wei Wang, Pei-bo He, Jie Lu, Chuan-xi Ma, Cheng Chang, Yong-Ling Ruan, Hai-ping Zhang

**Affiliations:** 1 Key Laboratory of Wheat Biology and Genetic Improvement on Southern Yellow and Huai River Valley, College of Agronomy, Anhui Agricultural University, Hefei 230036, China; 2 Innovation Cluster of Crop Molecular Biology and Breeding, Anhui Agricultural University, Hefei 230036, China; 3 State Key Laboratory for Crop Stress Resistance and High-Efficiency Production and College of Horticulture, Northwest A&F University, Yangling 712100, China; 4 Research School of Biology, The Australian National University, Canberra, ACT 2601, Australia

**Keywords:** wheat, low temperature, seed dormancy, pre-harvest sprouting, microRNA

## Abstract

In wheat, exposure to low temperatures (LTs) during the middle and late stages of seed development induces dormancy release; however, the underlying regulatory mechanism remains unclear. Here, using whole-transcriptome sequencing, we identified a novel microRNA (miR1832) that is downregulated by LT and located at a key node of the associated regulatory network. Germination assays showed that overexpression of miR1832 enhanced seed dormancy, whereas its silencing reduced seed dormancy. Sequence variation and association analyses further indicated that an A/G mutation at −670 bp in the miR1832 promoter is significantly associated with phenotypic variation in seed dormancy among wheat varieties, with the A allele correlated with strong dormancy. Through yeast one-hybrid, electrophoretic mobility shift assay, and dual-luciferase (LUC) reporter assays, we found that the LT-responsive Dof transcription factor TaDof-2D directly binds to the A site in the miR1832 promoter and inhibits its transcription. Subsequent expression analysis, dual-LUC assays, and 5′ rapid amplification of cDNA ends confirmed that miR1832 targets the *cytochrome P450* gene *TaP450-7A*, which is upregulated by LT and negatively regulates seed dormancy. Finally, physiological and biochemical analyses further demonstrated that the TaDof-2D–miR1832–TaP450-7A module participates in LT-induced dormancy release by modulating α-amylase activity and the abscisic acid and gibberellin pathways. These findings uncover a previously uncharacterized regulatory mechanism underlying LT-induced dormancy release and provide promising genetic resources and molecular markers for breeding wheat varieties with optimal dormancy levels.

## Teaser

This study reports the identification of the TaDof-2D–miR1832–TaP450-7A module as a key pathway mediating low-temperature-induced wheat seed dormancy release. The cleaved amplified polymorphic sequence (CAPS) marker 1832, developed based on the A/G polymorphism in the miR1832 promoter, provides a useful tool for breeding wheat varieties with enhanced pre-harvest sprouting resistance and improved adaptability to fluctuating climatic conditions.

## Introduction

As an adaptive trait, seed dormancy (SD) ensures that seeds germinate under favorable environmental conditions, thereby promoting species survival (Sato and Köhler, 2022). In agricultural production, adequate SD helps prevent pre-harvest sprouting (PHS) in major staple crops such as wheat and rice. PHS occurs when wheat and other cereal crops are exposed to wet conditions before or during harvest, resulting in considerable yield losses. Moreover, PHS negatively impacts grain quality through enzymatic degradation of starch and proteins and poses a serious food safety risk due to fungal contamination of germinated grains. Conversely, excessive SD can lead to delayed germination and uneven seedling emergence, which in turn negatively affect crop management and yield (Finkelstein et al., 2008; Liu et al., 2013, 2024; Xu et al., 2022). Therefore, it is critically important to unravel the molecular mechanisms underlying SD to develop wheat varieties with optimal dormancy levels that minimize PHS and other agronomic problems.

SD is a quantitative trait regulated by the interplay of endogenous and exogenous factors (Finch-Savage and Leubner-Metzger, 2006). As core endogenous regulators, the phytohormones abscisic acid (ABA) and gibberellic acid (GA) control the transition from SD to germination through their dynamic balance and complex interactions (Yan and Chen, 2020). Temperature, light, and nitrate availability represent the most important exogenous factors affecting the depth of SD. Upon perception of these signals, plants activate signaling interactions among endogenous phytohormones to regulate seed physiological processes (Seo et al., 2009). Among these environmental factors, temperature is particularly important (Chahtane et al., 2017), with elevated temperatures during seed maturation significantly reducing dormancy levels (Rodríguez et al., 2001; Jiménez et al., 2017). In wheat, sustained heat stress (temperatures >30°C for more than 12 days) during the middle and late stages of seed development significantly decreases dormancy, as dormancy is negatively correlated with the number of days >30°C (Biddulph et al., 2007). Similarly, our previous study demonstrated that high temperature treatment (35°C) from 21 to 35 days post-anthesis (DPA) significantly reduced dormancy by modulating the expression of key dormancy-related genes (e.g., *TaDOG1-3A*, *TaDOG1-3D*, and *TaMFT-3A*), as well as genes involved in ABA and GA signaling (e.g., *TaSnRK2*, *TaAFP*, and *TaGID*) (Jiang et al., 2023).

Interestingly, the effects of low temperature (LT) on SD differ markedly between the early and middle–late stages of grain development. Compared with normal temperature (NT, 25°C), LT treatment (13°C) after anthesis has been reported to enhance SD in both Shiroganekomugi (SK; weak dormancy) and Norin61 (strong dormancy) wheat cultivars, with the germination rate of SK reduced by 50% and Norin61 exhibiting complete inhibition of germination. Consistent with this enhanced dormancy, the wheat homolog of the dormancy-related gene *MOTHER OF FT AND TFL1* (*MFT*) is upregulated in dormant wheat seeds grown at LT (Nakamura et al., 2011). In contrast, exposure to LT (18°C/7.5°C) for one week during the middle to late stages of grain development induces the accumulation of large amounts of high–isoelectric point α-amylase, also known as late-maturity α-amylase (LMA), and promotes SD release. Under rainfall or other wet conditions, LMA promotes grain germination by degrading endosperm starch to supply energy to the developing plant (Mrva et al., 2006; Mares and Mrva, 2014; Derkx and Mares, 2020; Peery et al., 2023). At the molecular level, LMA is characterized by the transient expression of *α-amylase 1* (*α-Amy1*) genes on chromosome group 6. Specifically, one or more genes located on the long arm of chromosome 6B are critical for GA-induced α-amylase synthesis in the aleurone layer (Mrva and Mares, 1999; Barrero et al., 2013). Despite these advances, the detailed molecular mechanisms by which LT induces LMA expression remain poorly understood.

Non-coding RNAs do not encode proteins and include long non-coding RNAs (lncRNAs), short non-coding RNAs (such as rRNAs, tRNAs, and microRNAs [miRNAs]), and circular RNAs (circRNAs) (Rai et al., 2019; Wang et al., 2022). Widely participating in growth, development, and stress responses, miRNAs degrade or inhibit the translation of target mRNAs to suppress their expression (Cheng et al., 2021; Clepet et al., 2021; Zhang et al., 2022a; Jian et al., 2022; Shen et al., 2023; Lian et al., 2024). lncRNAs, which are non-coding RNA molecules longer than 200 nt, modulate gene expression at the transcriptional, post-transcriptional, and translational levels (Statello et al., 2021). The regulatory functions of circRNAs are mediated by the formation of R-loop structures, their roles as miRNA sponges, and/or their interactions with RNA polymerase II or RNA-binding proteins (Misir et al., 2022). Studies suggest that both miRNAs and lncRNAs participate in the regulation of SD, although the function of circRNAs in this context remains unknown. In rice, miR156 negatively regulates SD by promoting the expression of GA biosynthesis-related genes (e.g., *GNP1*, *SD1*, and *KAO*) and inhibiting the expression of GA metabolism-related genes (e.g., *GA2ox6*, *GA2ox8*, and *EUI1*) (Miao et al., 2019). In addition, the lncRNA VIVIpary promotes dormancy release and PHS by modulating ABA signaling and chromatin architecture (Yang et al., 2025). In wheat, miR9678 targets the lncRNA WSGAR to inhibit GA biosynthesis and promote GA catabolism, thereby positively regulating SD (Guo et al., 2018). Together, these findings highlight the importance of miRNAs and lncRNAs in regulating SD and indicate that GA and ABA are key players in this process. However, it remains unknown whether miRNAs, lncRNAs, and circRNAs participate in LT-mediated dormancy regulation in wheat.

In this study, we investigated the effects of LT during wheat development on SD and its underlying molecular regulatory mechanisms. We observed that LT treatment (10°C night/15°C day) significantly reduced dormancy levels in the wheat landrace Waitoubai (WTB) during the middle and late stages of seed development (21–35 DPA). Notably, exposure to LT significantly altered the expression of large numbers of mRNAs, miRNAs, lncRNAs, and circRNAs. Among these, a novel miRNA (unconservative_chr1A_1832; miR1832) was identified at a key node within the regulatory network and positively regulated SD in wheat, rice, and *Arabidopsis thaliana*. An A/G polymorphism in the miR1832 promoter was significantly associated with variation in SD phenotypes, with the A allele correlated with stronger dormancy. The DNA-binding with one finger (Dof) transcription factor (TF) TaDof-2D was found to bind directly to the 5′-AAGGC-3′ motif containing the A allele in the miR1832 promoter, thereby inhibiting miR1832 transcription. Furthermore, we demonstrated that miR1832 targets the *cytochrome P450* gene *TraesCS7A02G455300* (*TaP450-7A*) to regulate both ABA/GA balance and SD, and that *TaP450-7A* promotes SD release. TaDof-2D represses miR1832 expression, thereby relieving miR1832-mediated inhibition of *TaP450-7A*. Thus, we propose that the TaDof-2D–miR1832–TaP450-7A module mediates LT-induced dormancy release by mediating α-amylase activity and the ABA and GA pathways. These findings identify a novel regulatory module and provide promising gene targets and molecular markers for breeding wheat varieties with optimal dormancy levels.

## Results

### Low temperature (LT) during the mid-to-late stages of seed development induces dormancy release

**Figure 1:**
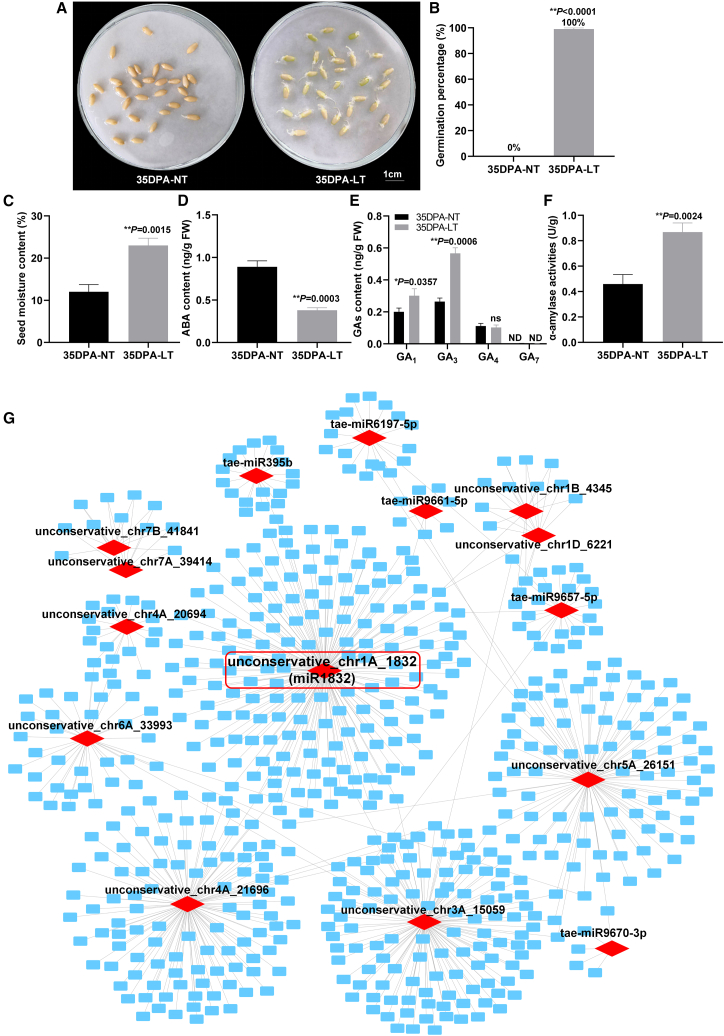
Germination percentages, physiological indicators, and regulatory networks of Waitoubai wheat seeds under low temperature. **(A)** Germination phenotypes of freshly harvested WTB seeds imbibed for 3 days. Seeds were collected at 35 days post-anthesis under normal-temperature (NT) and low-temperature (LT) conditions (35DPA-NT vs. 35DPA-LT). **(B)** Germination percentages of WTB seeds. **(C)** Moisture contents of WTB seeds. Values are means ± standard deviation (*n* = 3). **(D–F)** Contents of GAs and ABA and α-amylase activity in WTB seeds. GA_1_, gibberellin A_1_; GA_3_, gibberellin A_3_; GA_4_, gibberellin A_4_; GA_7_, gibberellin A_7_. Values are means ± standard deviation (*n* = 3). Statistical significance was analyzed using Student’s *t*-test (*∗P* < 0.05 and *∗∗P* < 0.01). **(G)** Regulatory network of mRNAs and miRNAs involved in LT-mediated seed dormancy. Red diamonds represent miRNAs, and blue boxes represent mRNAs. Detailed information on the mRNAs is provided in Supplemental Table 6.

To investigate the effect of LT exposure during the mid-to-late stages of seed development on SD, plants of the strong-dormancy wheat landrace WTB were exposed to either LT (15°C day/10°C night) or NT (25°C day/20°C night) from 21 to 35 DPA. Seeds were collected at 35 DPA to measure moisture content, which was 12% in NT-treated seeds and 23% in LT-treated seeds. LT-exposed seeds exhibited a germination percentage (GP) of 100%, whereas NT-exposed seeds showed 0% GP (Figure 1A–1C). To determine whether the change in SD phenotype was caused by LT itself or by differences in seed moisture content, LT-treated seeds were naturally air-dried to approximately 12% moisture content (Supplemental Figure 1A). Germination assays showed that the GP of air-dried LT-treated seeds remained at 100% (Supplemental Figure 1B and 1C), indicating that SD release is primarily driven by LT rather than increased moisture content. In addition, LT exposure significantly altered the ABA and GA contents as well as α-amylase activity in seeds. At 35 DPA, compared with NT-exposed seeds, LT-exposed seeds exhibited significantly increased GA_1_ and GA_3_ contents, reduced ABA content, and increased α-amylase activity (Figure 1D–1F). These results demonstrate that exposure to LT during the mid-to-late stages of seed development induces dormancy release.

### miR1832 plays a central role in regulating LT-induced dormancy release

To explore the molecular mechanism by which LT induces SD release, we performed whole-transcriptome sequencing on WTB seeds (without hulls) exposed to NT and LT conditions. A total of 4862 differentially expressed genes (DEGs) were identified between LT- and NT-exposed seeds, of which 4010 were upregulated and 852 were downregulated in LT-exposed seeds relative to NT-exposed seeds (Supplemental Figure 2A; Supplemental Table 1). Kyoto Encyclopedia of Genes and Genomes (KEGG) pathway enrichment analysis showed that these DEGs were significantly enriched in carbohydrate metabolism, energy metabolism, amino acid metabolism, signal transduction, and other biological processes (Supplemental Figure 2B). In addition, 82 miRNAs, 108 lncRNAs, and 9 circRNAs were differentially expressed between LT- and NT-exposed seeds (Supplemental Figure 2A; Supplemental Tables 2, 3, and 4). The expression patterns of several randomly selected DEGs, including 40 mRNAs, 10 miRNAs, 20 lncRNAs, and 5 circRNAs, were subsequently verified by real-time qPCR (Supplemental Figure 2C–2F; Supplemental Table 5), supporting the reliability of the transcriptomic sequencing data.

To elucidate functional relationships between miRNAs and their target genes involved in LT-induced dormancy release, we constructed a regulatory network based on differentially expressed mRNAs and miRNAs using the method described by Jiang et al. (2023). The predicted network revealed multiple potential one-to-one, one-to-many, and many-to-one regulatory relationships between miRNAs and mRNAs (Supplemental Table 6). Within this network, the novel miRNA unconservative_chr1A_1832 (designated miR1832) was located at a key node and was downregulated by LT (Figure 1G). The expression patterns of miR1832 under LT and NT conditions were verified by real-time qPCR, supporting its central role in regulating LT-induced dormancy release (Supplemental Figure 3A).

### miR1832 also regulates seed dormancy under normal temperature

Several dormancy-related genes, such as *DOG1* in *A. thaliana*, *TaMFT-3A* in wheat, and *SD6* in rice, are known to play important roles in regulating SD under both LT and NT conditions (Kendall et al., 2011; Nakamura et al., 2011; Graeber et al., 2014; Xu et al., 2022). To determine whether miR1832 is involved in SD regulation under NT conditions, we first examined its expression patterns at different seed developmental stages (21, 28, and 35 DPA, representing dormancy establishment), post-ripening stages (7, 14, and 21 days after harvest, representing dormancy release), and imbibition stages (1, 6, 9, 12, and 36 h, also representing dormancy release) in two strong-dormancy varieties (WTB and Hongmanchun21 [HMC21]) and two weak-dormancy varieties (Zhongyou9507 [ZY9507] and Jing411 [J411]). Overall, miR1832 expression increased during dormancy establishment and decreased during dormancy release. Notably, miR1832 expression was consistently higher in the strong-dormancy varieties WTB and HMC21 than in the weak-dormancy varieties ZY9507 and J411 (Supplemental Figure 3B–3D). Together, these results suggest that miR1832 contributes to the establishment and maintenance of SD under NT conditions.

**Figure 2:**
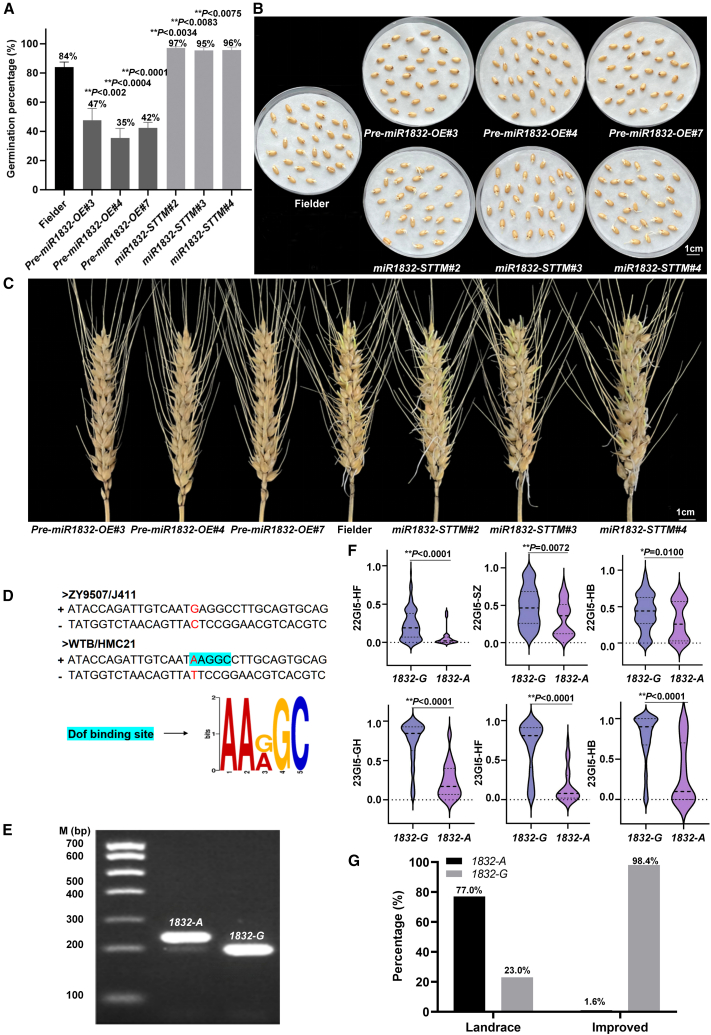
miR1832 positively regulates seed dormancy in wheat. **(A)** Germination percentages of Fielder, *pre-miR1832-OE*, and *miR1832-STTM* wheat seeds imbibed for 3 days. Values are means ± standard deviation (*n* = 3). Statistical significance was analyzed using Student’s *t*-test (*∗∗P* < 0.01). **(B and C)** Germination phenotypes of Fielder, *pre-miR1832-OE*, and *miR1832-STTM* wheat seeds imbibed for 3 days (B) and wheat spikes imbibed for 5 days (C). **(D)** miR1832 sequence comparison between weak-dormancy varieties (Zhongyou9507 [ZY9507] and Jing411 [J411]) and strong-dormancy varieties (Waitoubai [WTB] and Hongmangchun21 [HMC21]). The cyan box indicates the Dof-binding site, and red bases indicate the mutation position. **(E)** Detection of the cleaved amplified polymorphic sequence (CAPS) marker 1832 for miR1832 using 2.0% agarose gel electrophoresis. **(F)** Differences in germination indices of 22GI5-HF, 22GI5-SZ, 22GI5-HB, 23GI5-GH, 23GI5-HF, and 23GI5-HB among 160 wheat varieties carrying the two allelic variants (*1832-A* and *1832-G*) of marker 1832. *1832-A/G* indicate CAPS marker alleles corresponding to the A/G nucleotide mutation at −670 bp in the miR1832 promoter. Statistical significance was analyzed using Student’s *t*-test (*∗P* < 0.05 and *∗∗P* < 0.01). **(G)** miR1832 allele frequencies in landraces and improved varieties.

To test the regulatory role of miR1832 in wheat SD, we overexpressed its precursor and silenced its mature transcript in wild-type (WT) Fielder wheat. Three overexpression lines (*pre-miR1832-OE#3*, *pre-miR1832-OE#4*, and *pre-miR1832-OE#7*) and three short tandem target mimic (STTM) lines (*miR1832-STTM#2*, *miR1832-STTM#3*, and *miR1832-STTM#4*) were generated. Compared with Fielder, miR1832 expression was significantly upregulated in the *pre-miR1832-OE#3/4/7* lines but significantly downregulated in the *miR1832-STTM#2/3/4* lines (Supplemental Figure 4A). The GPs of the three overexpression lines (*pre-miR1832-OE#3* [47%], *pre-miR1832-OE#4* [35%], and *pre-miR1832-OE#7* [42%]) were significantly lower than that of Fielder (84%), whereas the GPs of the three (Short Tandem Target Mimic) STTM lines (*miR1832-STTM#2* [97%], *miR1832-STTM#3* [95%], and *miR1832-STTM#4* [96%]) were higher (Figure 2A–2C). These results confirm that miR1832 positively regulates wheat SD.

Homologous SD-related genes often maintain conserved functions across plant species, such as *AtABI3* in *Arabidopsis thaliana* (*A. thaliana)* and *TaVp-1* in wheat (Yang et al., 2007), *DOG1* in *A. thaliana* and *TaDOG1L1* in wheat (Teng et al., 2008; Ashikawa et al., 2014), *OsSdr4* in rice and *TaSdr* in wheat (Zhang et al., 2014), *HvMKK3* in barley and *TaMKK3-A* in wheat (Nakamura et al., 2016; Torada et al., 2016), *HvQsd1* in barley and *TaQsd1* in wheat (Wei et al., 2019), and *OsSD6* in rice and *TaSD6* in wheat (Xu et al., 2022). Sequence alignment showed that wheat-derived miR1832 shares high similarity with its rice homolog OsmiR1832 (89%) and *A. thaliana* homolog AtmiR1832 (78%) (Supplemental Figure 5A). To investigate whether miR1832, OsmiR1832, and AtmiR1832 share conserved roles in SD regulation, we heterologously overexpressed the miR1832 precursor in rice and *A. thaliana* and silenced the mature transcripts of OsmiR1832 and AtmiR1832 via STTM (Supplemental Figure 4B and 4C). The GPs of miR1832-overexpressing rice and *A. thaliana* seeds were significantly lower than those of WT Nip and Col-0, respectively, whereas the GPs of OsmiR1832 and AtmiR1832 STTM seeds were significantly higher (Supplemental Figures 5B, 5C, and 6). These results suggest that miR1832 enhances SD across plant species and that OsmiR1832 and AtmiR1832 share similar functions with miR1832 in wheat.

### The A/G polymorphism in the miR1832 promoter is significantly associated with phenotypic variation in seed dormancy

To explore the molecular basis underlying miR1832-mediated phenotypic differences in SD (Supplemental Figure 3B and 3D), we first cloned the 1,000 bp upstream promoter and precursor sequences of miR1832 from the strong-dormancy landraces WTB and HMC21 as well as from the weak-dormancy varieties ZY9507 and J411. miR1832 was found to possess a typical stem–loop structure, and sequence analysis revealed two single-nucleotide polymorphisms (SNPs) in the miR1832 promoter and precursor between the strong-dormancy landraces (WTB and HMC21) and weak-dormancy varieties (ZY9507 and J411) (Supplemental Figure 7A). Promoter *cis*-element prediction analysis indicated that the A-to-G mutation at −670 bp in the miR1832 promoter may lead to deletion of a Dof-binding site in the weak-dormancy varieties J411 and ZY9507 (Figure 2D and Supplemental Figure 7A). By contrast, the T-to-C mutation at +156 bp in the precursor did not alter the stem–loop structure (Supplemental Figure 7B).

To determine whether the A/G mutation at −670 bp in the miR1832 promoter influences SD phenotypes, we developed a cleaved amplified polymorphic sequence (CAPS) marker (1832) based on this mutation (Figure 2E) and used it to genotype 160 wheat varieties (160 WVs) exhibiting varying dormancy levels. The two alleles of marker 1832 were designated *1832-A* and *1832-G*. Mann–Whitney *U*-test results revealed significant differences in germination index (GI) values between varieties carrying the *1832-A* allele and those carrying the *1832-G* allele (Figure 2F; Supplemental Tables 7 and 8). The *1832-A* allele was significantly associated with low GI (corresponding to strong dormancy and PHS resistance), whereas *1832-G* was significantly associated with high GI (corresponding to weak dormancy and PHS susceptibility) (*P* < 0.01). To further validate the association between the A/G mutation and SD phenotypes, we examined miR1832 expression in 10 wheat varieties carrying the A allele and 10 varieties carrying the G allele. miR1832 expression was significantly higher in varieties with the A allele than in those with the G allele (Supplemental Figure 7C; Supplemental Table 7). Collectively, these results indicate that the A/G mutation in the miR1832 promoter is significantly associated with phenotypic variation in SD, with the A allele associated with strong dormancy and PHS resistance.

To investigate whether the favorable *1832-A* allele associated with strong dormancy and PHS resistance has been selected for or against during wheat breeding, we genotyped 124 landraces and 245 improved varieties using the 1832 marker. Among the 124 landraces, the frequency of *1832-A* (76.6%, *n* = 95) was significantly higher than that of *1832-G* (23.4%, *n* = 29). By contrast, among the 245 improved varieties, only 4 (1.6%) carried the *1832-A* allele, whereas 241 (98.4%) carried *1832-G* (Figure 2G; Supplemental Table 9). These results suggest that the *1832-A* allele has not been fully utilized in modern wheat breeding and may represent a valuable target for improving PHS resistance through molecular design breeding.

### TaDof-2D directly binds the A site in the miR1832 promoter to repress its transcription

The A site at −670 bp in the miR1832 promoter of the strong-dormancy landraces WTB and HMC21 was predicted to be a Dof TF binding site. To identify upstream TFs capable of binding to this site, we amplified two 45-bp fragments flanking the −670 bp region, containing either the A or G allele, designated miR1832-A and miR1832-G, respectively. The miR1832-A fragment was cloned into the pAbAi vector to generate a bait for yeast one-hybrid (Y1H) screening. A total of 35 positive clones were identified (Supplemental Table 10), including three Dof TFs: TaDof-2D (*TraesCS2D02G100300*), TaDof-3A (*TraesCS3A02G180600*), and TaDof-4D (*TraesCS4D02G285100*). Based on transcriptome analysis of WTB wheat seeds under LT and NT, *TaDof-2D* expression was significantly upregulated by LT, whereas *TaDof-3A* and *TaDof-4D* expression was not (Supplemental Table 1; Supplemental Figure 8A).

**Figure 3:**
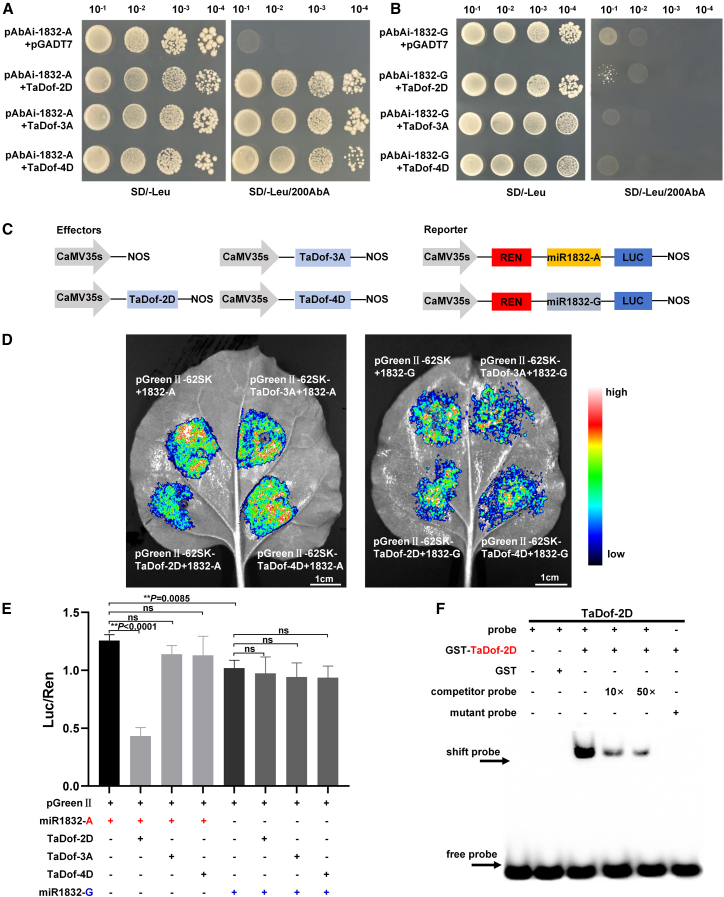
TaDof-2D inhibits miR1832 transcription. **(A)** Yeast one-hybrid (Y1H) assay showing that TaDof-2D, TaDof-3A, and TaDof-4D bind to the miR1832 promoter containing the A allele. SD/−Leu, synthetic dropout medium lacking leucine. AbA, aureobasidin A. **(B)** Y1H assay showing that TaDof-2D, TaDof-3A, and TaDof-4D fail to bind to the miR1832 promoter containing the G allele. **(C)** Schematic structures of reporter genes and effector constructs used in the dual-luciferase (LUC) assay. **(D and E)** Transient dual-LUC reporter assay showing that TaDof-2D inhibits the activity of the miR1832 promoter containing the A allele. Representative LUC signals in tobacco leaves **(D)** and LUC/*Renilla* LUC (REN) ratios **(E)** are shown. Values are means ± standard deviation (*n* = 3). Statistical significance was analyzed using Student’s *t*-test (*∗∗P* < 0.01). ns, not significant. **(F)** Electrophoretic mobility shift assay showing binding of TaDof-2D to the Dof-binding motif (AAGGC) in the miR1832 promoter. Ten- and 50-fold unlabeled probes were added as competitors. miR1832-A, A-allele-carrying miR1832 promoter fragment; miR1832-G, G-allele-carrying miR1832 promoter fragment; GST, glutathione S-transferase.

Y1H assays revealed that TaDof-2D, TaDof-3A, and TaDof-4D bind to miR1832-A but not to miR1832-G (Figure 3A and 3B and Supplemental Figure 8B–8D). Dual-luciferase (LUC) reporter assays further demonstrated allele-specific regulation: only TaDof-2D significantly reduced the transcriptional activity of the miR1832 promoter carrying the A allele, whereas none of the three TFs affected the G-allele-containing promoter (Figures 3C–3E). Consistent with this, the transcriptional activity of the A-allele promoter was significantly higher than that of the G-allele promoter (Figure 3E), consistent with the higher miR1832 expression observed in wheat varieties carrying the A allele (Supplemental Figure 3B–3D). To determine whether this regulation is direct, electrophoretic mobility shift assays (EMSAs) were performed, revealing that only TaDof-2D specifically binds to the 5′-AAGGC-3′ motif containing the A allele, whereas no binding was detected between the G-allele-containing motif (miR1832-G) and TaDof-2D, TaDof-3A, or TaDof-4D (Figure 3F and Supplemental Figure 8E and 8F). Collectively, these results establish that TaDof-2D directly binds to the A site in the miR1832 promoter to repress its expression. Given that LT upregulated *TaDof-2D* expression but repressed miR1832 expression in WTB seeds (Supplemental Table 1; Supplemental Figure 8A), we propose that LT-induced upregulation of TaDof-2D suppresses miR1832 expression, thereby promoting dormancy release.

**Figure 4:**
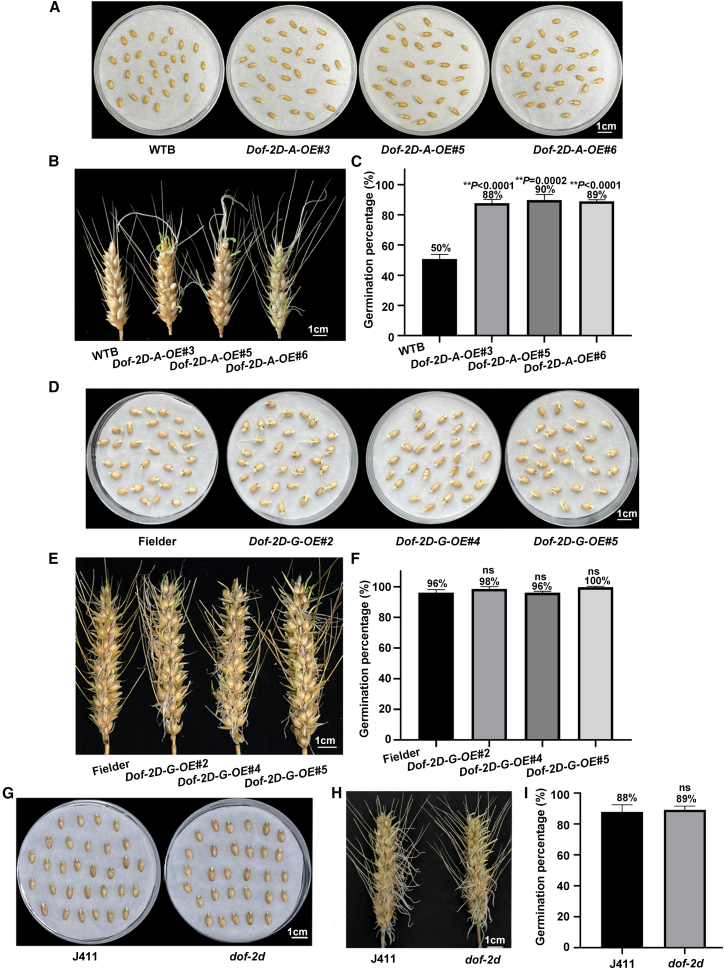
TaDof-2D regulates wheat seed dormancy by binding to the A site in the miR1832 promoter. **(A and B)** Germination phenotypes of Waitoubai (WTB) and *Dof-2D-A-OE#3/5/6* seeds imbibed for 3 days (A) and spikes imbibed for 5 days (B). **(C)** Germination percentages of WTB and *Dof-2D-A-OE#3/5/6* seeds. Values are means ± standard deviation (*n* = 3). **(D and E)** Germination phenotypes of Fielder and *Dof-2D-G-OE#2/4/5* seeds imbibed for 3 days (D) and spikes imbibed for 5 days (E). **(F)** Germination percentages of Fielder and *Dof-2D-G-OE#2/4/5* seeds. Values are means ± standard deviation (*n* = 3). **(G and H)** Germination phenotypes of Jing411 (J411) and *dof-2d* mutant seeds imbibed for 3 days (G) and spikes imbibed for 5 days (H). **(I)** Germination percentages of J411 and *dof-2d* seeds. Values are means ± standard deviation (*n* = 3). Statistical significance was analyzed using Student’s *t*-test (*∗∗P* < 0.01). ns, not significant.

To confirm the role of *TaDof-2D* in wheat SD, we overexpressed the coding sequence (CDS) of *TaDof-2D* in wheat varieties Fielder (carrying the G allele) and WTB (carrying the A allele). Three overexpression lines in the Fielder background (*Dof-2D-G-OE#2*, *Dof-2D-G-OE#4*, and *Dof-2D-G-OE#5*) and three overexpression lines in the WTB background (*Dof-2D-A-OE#3*, *Dof-2D-A-OE#5*, and *Dof-2D-A-OE#6*) were generated (Supplemental Figure 9A and 9B). Germination tests showed that *Dof-2D-A-OE#3/5/6* exhibited significantly higher GP values (indicative of reduced dormancy) compared with WT WTB, whereas no significant differences in GP were observed between *Dof-2D-G-OE#2/4/5* and WT Fielder (Figure 4A–4F). Additionally, we obtained a *TaDof-2D* ethyl methane sulfonate (EMS) mutant (designated *dof-2d*) in the J411 background (carrying the G allele), which harbors a SNP mutation (G/A) at the 382nd amino acid, resulting in premature termination of translation (Supplemental Figure 9C). The GP of *dof-2d* was not significantly different from that of WT J411 (Figure 4G–4I). Consistent with these phenotypes, miR1832 expression was significantly downregulated in the *Dof-2D-A-OE#3/5/6* lines compared with WT WTB (Supplemental Figure 9D), whereas no significant differences were observed between *Dof-2D-G-OE#2/4/5* and Fielder or between *dof-2d* and J411 (Supplemental Figure 9E and 9F). We further examined the expression profiles of *TaDof-2D* and miR1832 in the roots, stems, leaves, spikes, and seeds of WTB by RT–qPCR. *TaDof-2D* expression was relatively high in roots and stems but low in leaves, spikes, and seeds, whereas miR1832 showed the opposite pattern (Supplemental Figure 9G and 9H). Together, these findings demonstrate that *TaDof-2D* specifically binds to the A site in the miR1832 promoter to repress its transcription, thereby reducing SD in wheat.

### *TaP450-7A* is a target of miR1832

**Figure 5:**
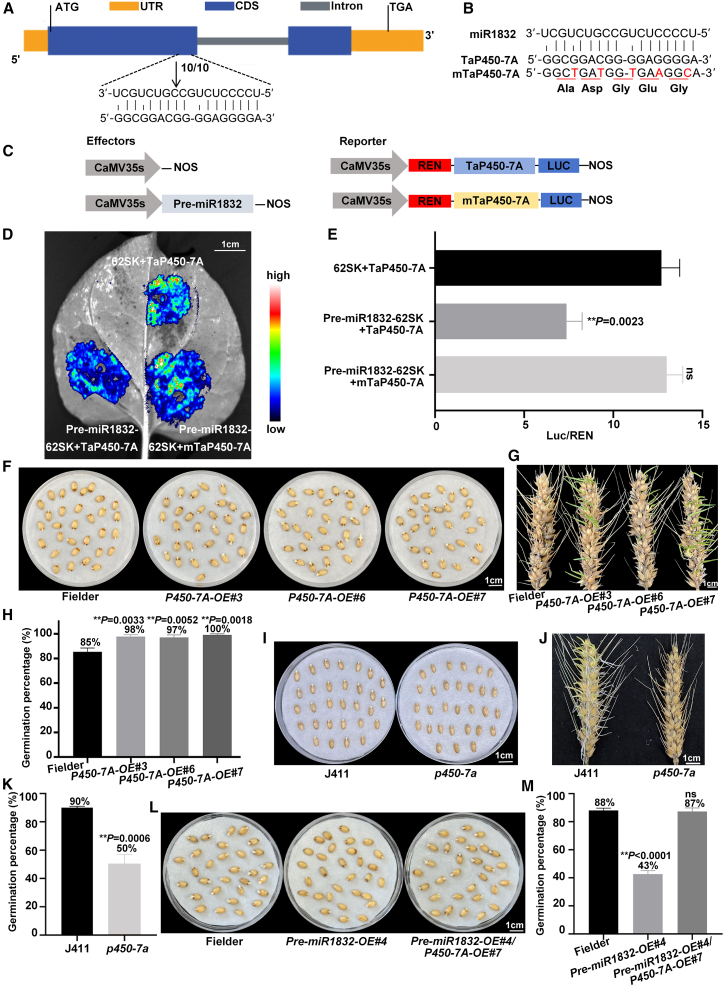
Validated targets and functional implications of miR1832. **(A)** 5′ rapid amplification of cDNA ends assay showing the miR1832-mediated cleavage site in *TaP450-7A*. Yellow, blue, and gray boxes represent untranslated regions (UTRs), exons, and introns, respectively. **(B)** Schematic diagram of the miR1832-resistant version (mTaP450-7A) generated by introducing synonymous mutations into *TaP450-7A*. **(C)** Schematic structures of reporter genes and effector elements used in the dual-luciferase (LUC) assay. CaMV35S, cauliflower mosaic virus 35S promoter; NOS, nopaline synthase. **(D)** LUC activity in *N. benthamiana* tissues transformed with reporter and effector/reporter constructs. **(E)** LUC assays showing the targeting relationship between miR1832 and *TaP450-7A* in *N. benthamiana*. Values are means ± standard deviation (*n* = 3). **(F and G)** Germination phenotypes of Fielder and *P450-7A-OE#3/6/7* seeds imbibed for 3 days **(F) and spikes imbibed for 5 days (G)**. **(H)** Germination percentages of Fielder and *P450-7A-OE#3/6/7* seeds. Values are means ± standard deviation (*n* = 3). **(I and J)** Germination phenotypes of Jing411 (J411) and *p450-7a* mutant seeds imbibed for 3 days **(I) and spikes imbibed for 5 days (J)**. **(K)** Germination percentages of J411 and *p450-7a* seeds. Values are means ± standard deviation (n = 3). **(L)** Germination phenotypes of Fielder, *pre-miR1832-OE#4*, and *pre-miR1832-OE#4/P450-7A-OE#7* seeds imbibed for 3 days. Values are means ± standard deviation (*n* = 3). **(M)** Germination percentages of Fielder, *pre-miR1832-OE#4*, and *pre-miR1832-OE#4/P450-7A-OE#7* seeds. Values are means ± standard deviation (*n* = 3). Statistical significance was analyzed using Student’s *t*-test (*∗∗P* < 0.01). ns, not significant.

To identify genes targeted by miR1832, we combined transcriptome sequencing with psRNATarget prediction. Three potential targets were identified: the ethylene-responsive TF *TaERF-1D* (*TraesCS1D02G221500*), the NAC (NAM, ATAF, and CUC) TF *TaNAC-4A* (*TraesCS4A02G219700*), and the cytochrome P450 gene *TaP450-7A* (*TraesCS7A02G455300*). Contrary to miR1832, all three genes were significantly upregulated in LT-exposed WTB seeds (Supplemental Figure 10A and 10B). We further examined their expression patterns in freshly harvested *pre-miR1832-OE*, *miR1832-STTM*, and WT Fielder seeds. Compared with Fielder, *TaP450-7A* expression was significantly reduced in *pre-miR1832-OE* seeds but increased in *miR1832-STTM* seeds. By contrast, no significant differences were observed in the expression of *TaERF-1D* or *TaNAC-4A* between *pre-miR1832-OE* or *miR1832-STTM* lines and Fielder (Supplemental Figure 10C–10E). These results suggest that *TaP450-7A* is a likely target gene of miR1832. To confirm direct targeting, we performed a 5′ rapid amplification of cDNA ends (5′-RACE) assay to map the miR1832-directed cleavage site within *TaP450-7A* mRNA (Figure 5A). To further verify miR1832-mediated regulation of *TaP450-7A in vivo*, we introduced synonymous mutations into *TaP450-7A* to generate a miR1832-resistant version (mTaP450-7A) (Figure 5B), which can no longer be targeted and cleaved by miR1832. Dual-LUC assays in *Nicotiana benthamiana* (*N. benthamiana*) epidermal cells showed that miR1832 expression significantly inhibited TaP450-7A-LUC reporter activity, whereas mTaP450-7A-LUC reporter activity was not significantly affected (Figure 5C–5E). RT-qPCR analysis further showed that *TaP450-7A* expression was significantly lower in wheat varieties carrying the *miR1832-A* allele than in those carrying the *miR1832-G* allele (Supplemental Figure 10F). Together, these results demonstrate that miR1832 directly targets and cleaves *TaP450-7A* mRNA *in vivo*.

To confirm the role of *TaP450-7A* in wheat SD, we generated three overexpression lines (*P450-7A-OE#3*, *P450-7A-OE#6*, and *P450-7A-OE#7*) in the Fielder background. *TaP450-7A* expression was significantly higher in *P450-7A-OE#3*/6/7 than in Fielder (Supplemental Figure 11A), and the GPs of these overexpression lines (*P450-7A-OE#3* [98%], *P450-7A-OE#6* [97%], and *P450-7A-OE#7* [100%]) were higher than that of Fielder (85%) (Figure 5F–5H). Additionally, we obtained a *TaP450-7A* EMS mutant (named *p450-7a*) in the J411 background, which harbors a SNP mutation (G/A) at the 19th amino acid, resulting in premature termination of translation (Supplemental Figure 11B). The GP of *p450-7a* (50%) was significantly lower than that of WT J411 (90%) (Figure 5I–5K). Overall, these results indicate that *TaP450-7A* negatively regulates SD, whereas miR1832 positively regulates SD, consistent with a miR1832–*TaP450-7A* regulatory module.

To investigate the genetic relationship between miR1832 and *TaP450-7A*, we further generated hybrid lines overexpressing both miR1832 and *TaP450-7A* (*pre-miR1832-OE#4/P450-7A-OE#7*). These hybrid lines reversed the strong dormancy phenotype of miR1832-overexpression lines, restoring SD levels to those of WT plants (Figure 5L and 5M). We also examined *TaP450-7A* expression in different *TaDof-2D* transgenic lines. *TaP450-7A* expression was significantly upregulated in *Dof-2D-A-OE#3/5/6* compared with WT WTB (Supplemental Figure 10G), whereas no significant differences were observed between *Dof-2D-G-OE#2/4/5* and WT Fielder or between *dof-2d* and WT J411 (Supplemental Figure 10H and 10I). These observations suggest that *TaDof-2D* binds specifically to the A site in the miR1832 promoter in *Dof-2D-A-OE#3/5/6* lines, repressing miR1832 expression and thereby upregulating *TaP450-7A*, ultimately reducing SD.

### The TaDof-2D–miR1832–TaP450-7A module likely promotes LT-induced dormancy release by modulating α-amylase activity and ABA/GA pathways

Exposure to LT during the middle and late stages of wheat seed development induces the expression of LMA genes, thereby promoting germination under high-humidity conditions (Derkx and Mares, 2020; Peery et al., 2023). In addition, GA and ABA antagonistically modulate the expression of LMA genes (Jacobsen, 1973; Higgins et al., 1976; Mrva and Mares, 2001; Zhang et al., 2022b). We therefore hypothesized that ABA, GA, and α-amylase (including LMA) are jointly involved in LT-induced dormancy release in WTB seeds. Compared with NT-exposed WTB seeds, the expression levels of six LMA genes (*TraesCS6A02G334200*, *TraesCS6B02G349700*, *TraesCS6B02G349500*, *TraesCS6B02G364900*, *TraesCS6B02G349800*, and *TraesCS6B02G364800*) were significantly upregulated in LT-exposed WTB seeds, whereas two α-amylase inhibitor genes (*TaCM1-7D* and *TaCM2-7B*) were significantly downregulated. In addition, four genes related to GA signaling (*TaGID1-1B*, *TaGID1-1D*, *TaGASR7-7B*, and *TaGASR7-7D*) and two genes related to ABA catabolism (*TaABA8′OH2-5B* and *TaABA8′OH2-5D*) were significantly upregulated in LT-exposed WTB seeds, whereas two genes related to ABA signaling (*TaABI5-3B* and *TaABI5-3D*) were significantly downregulated (Supplemental Figure 12). The expression patterns of these genes were verified by RT–qPCR (Supplemental Table 11). LT-exposed WTB seeds exhibited significantly higher GA_1_ and GA_3_ contents and α-amylase activity, but significantly lower ABA content, than NT-exposed WTB seeds (Figure 1D–1F). Collectively, these results suggest that exposure to LT during the middle and late stages of seed development induces dormancy release by modulating LMA accumulation as well as GA and ABA biosynthesis, catabolism, and signaling.

**Figure 6:**
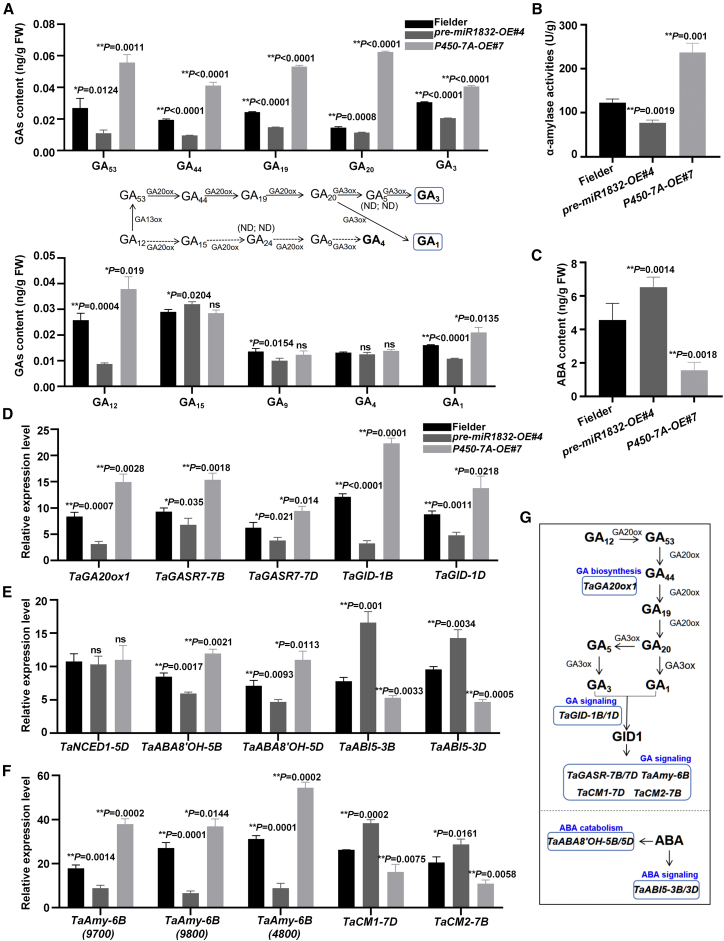
The miR1832–TaP450-7A module promotes LT-induced dormancy release by influencing α-amylase activity and the ABA and GA pathways. **(A)** GA contents in seeds of Fielder, *pre-miR1832-OE#4*, and *P450-7A-OE#7* imbibed for 24 h, with a schematic illustration of two major GA biosynthetic pathways: the non-13-hydroxylation pathway (including GA_15_ [gibberellin A_15_], GA_9_ [gibberellin A_9_], GA_24_ [gibberellin A_24_], and GA_4_ [gibberellin A_4_]) and the early 13-hydroxylation pathway (comprising GA_53_ [gibberellin A_53_], GA44 [gibberellin A_44_], GA_19_ [gibberellin A_19_], GA_20_ [gibberellin A_20_], GA_1_ [gibberellin A_1_], GA_5_ [gibberellin A_5_], and GA_3_ [gibberellin A_3_]). FW, fresh weight; ox, oxidase; ND, not detected. Values are means ± standard deviation (*n* = 3). **(B and C)** ABA content and α-amylase activity in seeds of Fielder, *pre-miR1832-OE#4*, and *P450-7A-OE#7* imbibed for 24 h. FW, fresh weight. Values are means ± standard deviation (*n* = 3). **(D–F)** Expression levels of key genes related to α-amylase biosynthesis and ABA and GA signaling in seeds of Fielder, *pre-miR1832-OE#4*, and *P450-7A-OE#7* imbibed for 24 h. Gene IDs: *TraesCS5D02G038800* (*TaNCED1*), *TraesCS5B02G236500* (*TaABA8′OH-5B*), *TraesCS5D02G244900* (*TaABA8′OH-5D*), *TraesCS3B02G404400* (*TaABI5-3B*), *TraesCS3D02G364900* (*TaABI5-3D*), *TraesCS3D02G393900* (*TaGA20ox1*), *TraesCS7B02G115300* (*TaGASR7-7B*), *TraesCS7A02G208100* (*TaGASR7-7A*), *TraesCS1B02G265900* (*TaGID1-1B*), *TraesCS1D02G254500* (*TaGID1-1D*), *TraesCS6B02G349700* (*TaAmy1-6B*), *TraesCS6B02G349800* (*TaAmy1-6B*), *TraesCS6B02G364800* (*TaAmy1-6B*), *TraesCS7D02G168000* (*TaCM1-7D*), and *TraesCS7B02G072000* (*TaCM2-7B*). Values are means ± standard deviation (*n* = 3). **(G)** Chromosomal locations of key genes involved in GA and ABA biosynthesis, metabolism, and signal transduction pathways. Values are means ± standard deviation (*n* = 3). Statistical significance was analyzed using Student’s *t*-test (*∗P* < 0.05 and *∗∗P* < 0.01). ns, not significant.

To further examine whether the TaDof-2D–miR1832–TaP450-7A module promotes LT-induced dormancy release by modulating key components of the GA and ABA signaling pathways, we measured GA and ABA contents as well as α-amylase activity in *pre-miR1832-OE#4*, *P450-7A-OE#7*, and WT Fielder seeds imbibed for 24 h. Two major GA biosynthetic pathways were detected: the non-13-hydroxylation pathway (including GA_15_, GA_9_, GA_24_, and GA_4_) and the early 13-hydroxylation pathway (comprising GA_53_, GA_44_, GA_19_, GA_20_, GA_1_, GA_5_, and GA_3_). Among these, the contents of key intermediates (GA_53_, GA_44_, GA_19_, and GA_20_) and bioactive GAs (GA_1_ and GA_3_) in the early 13-hydroxylation pathway were significantly increased in *TaP450-7A-OE#7* seed but significantly decreased in *pre-miR1832-OE#4* seeds (Figure 6A). In addition, *TaP450-7A-OE#7* seeds showed significantly decreased ABA content and increased α-amylase activity, whereas *pre-miR1832-OE#4* seeds exhibited the opposite trend (Figure 6B and 6C). These data provide strong evidence that the miR1832–TaP450-7A module regulates SD by influencing the balance between ABA and GA. We next analyzed the expression patterns of genes involved in ABA and GA pathways in *pre-miR1832-OE#4*, *P450-7A-OE#7*, and Fielder seeds imbibed for 24 h. The expression levels of genes related to GA biosynthesis (*TaGA20ox1*), GA signaling (*TaGASR7-7B*, *TaGASR7-7D*, *TaGID1-1B*, and *TaGID-1D*), and ABA catabolism (*TaABA8′OH-5B* and *TaABA8′OH-5D*) were significantly lower in *pre-miR1832-OE#4* seeds than in Fielder seeds. By contrast, genes related to ABA signaling (*TaABI5-3B* and *TaABI5-3D*) showed significantly higher expression in *pre-miR1832-OE#4* seeds. Furthermore, expression of α-amylase genes *TaAmy1-6B* (*TaAmy-6B-9700*, *TaAmy-6B-9800*, and *TaAmy-6B-4800*) was significantly lower in *pre-miR1832-OE#4* seeds, whereas the expression levels of α-amylase inhibitor genes (*TaCM1-7D* and *TaCM2-7B*) were significantly higher than in Fielder seeds. Compared with *pre-miR1832-OE#4* seeds, *P450-7A-OE#7* seeds exhibited opposite expression patterns (Figure 6D–6F). Collectively, these results suggest that the TaDof-2D–miR1832–TaP450-7A module mediates LT-induced dormancy release by modulating α-amylase activity and the GA/ABA pathways.

## Discussion

### miR1832 represses seed dormancy release under LT and NT conditions

Here, we observed that exposure to LT during the mid-to-late stages of seed development promotes dormancy release and reduces dormancy levels in the wheat landrace WTB. Whole-transcriptome sequencing revealed large numbers of coding and non-coding RNAs involved in LT-induced dormancy release. In particular, we identified a novel miRNA (miR1832) as a key node in the LT-response network that is downregulated by LT. Under NT conditions, overexpression of miR1832 significantly enhanced wheat SD, whereas silencing of miR1832 promoted dormancy release and germination. These findings demonstrate that miR1832 functions as an important regulator of SD under both LT and NT conditions. To date, no miRNAs have been reported to be involved in LT-induced dormancy release during seed development in wheat. Thus, the identification of miR1832 in this study helps clarify the molecular mechanisms underlying miRNA-mediated regulation of wheat SD under LT and NT conditions. Interestingly, we also found that tae-miR408, which shares 95% sequence identity with the germination-related miR408 in *A. thaliana* (Jiang et al., 2021), was significantly downregulated by LT (Supplemental Table 2; Supplemental Figure 13A and 13B). Therefore, we propose that tae-miR408 may also be involved in LT-induced dormancy release and that a close regulatory relationship may exist between miR1832 and tae-miR408.

### TaDof-2D acts as an upstream temperature-responsive repressor of miR1832

The A-to-G mutation at −670 bp in the miR1832 promoter leads to deletion of a Dof TF binding site in the weak-dormancy varieties J411 and ZY9507, and the Dof TF TaDof-2D was identified as an upstream regulator capable of binding to the A site in the miR1832 promoter based on Y1H screening, expression analysis, EMSA, and dual-LUC reporter assays. *TaDof-2D* expression was upregulated by LT and negatively influenced SD, in contrast to miR1832. Dof domain proteins are plant-specific TFs with a highly conserved DNA-binding domain that generally contains a single C2–C2 zinc finger (Yanagisawa, 2002; 2004). To date, only a few *Dof* genes have been reported to be involved in SD regulation. For example, DOF AFFECTING GERMINATION 1 (DAG1) acts as a repressor of seed germination in *A. thaliana.* Specifically, seeds of the *dag1*-knockout mutant are non-dormant and can germinate in the dark (Papi et al., 2000; Gabriele et al., 2010). In *A. thaliana*, DOF6 negatively regulates seed germination by interacting with TCP14, a positive regulator of seed germination, and by influencing the expression of a specific set of ABA-related genes (Rueda-Romero et al., 2012). Furthermore, DOF6 interacts with the DELLA protein RGA-LIKE2 (RGL2), which is involved in GA signaling. The RGL2–DOF6 complex activates the expression of *GATA12*, which encodes a GATA-type zinc-finger TF, thereby enforcing primary SD in *A. thaliana* (Ravindran et al., 2017). These findings highlight the important roles of Dof family members in SD regulation. Phylogenetic analysis suggests that *TaDof-2D* has a relatively distant relationship to previously characterized Dof genes, suggesting that it represents a novel, potentially wheat-specific, member of the Dof gene family (Supplemental Figure 13C).

### miR1832 targets *TaP450-7A* to suppress LT-induced dormancy release

miRNAs often exert their roles in development by degrading target mRNAs. Here, we identified *TaP450-7A* as a target gene of miR1832 based on a combination of computational prediction, expression analysis, 5′-RACE, and LUC reporter assays. Unlike miR1832, *TaP450-7A* was upregulated by LT and negatively regulated wheat SD. Cytochrome P450 (CYP450) proteins constitute the largest enzyme family in plants (Hansen et al., 2021; Krishnamurthy et al., 2021) and are known to regulate phytohormones, development, and stress responses (Wan et al., 2013; Ma et al., 2015b; Tamiru et al., 2015; Kato et al., 2020; Huang et al., 2024; Sun et al., 2024). To date, three *CYP450* genes have been reported to participate in SD- and germination-related processes. The *CYP707A* family genes *CYP707A1* and *CYP707A2* promote ABA metabolism and GA biosynthesis through the ABA 8′-hydroxylation pathway, thereby affecting SD and germination in *A. thaliana* (Kushiro et al., 2004; Okamoto et al., 2006). Additionally, He et al. (2019) reported that *CYP72A9* regulates the homeostasis of bioactive GA_4_ in the developing seeds and siliques of *A. thaliana*, and its disruption significantly reduces primary SD by increasing GA_4_ levels. Phylogenetic analysis suggests that *TaP450-7A* represents a novel member of the CYP450 family associated with SD under LT and NT conditions (Supplemental Figure 13D). This gene provides a valuable entry point for further dissection of the molecular regulatory network underlying SD in wheat.

### The TaDof-2D–miR1832–TaP450-7A module regulates LT-induced dormancy release

In wheat, exposure to LT during the middle and late stages of seed development induces the expression of high-isoelectric-point LMA, thereby promoting germination under high-humidity conditions. Previous studies suggested that GA and ABA are involved in LT-induced LMA expression, but the precise regulatory mechanisms remain unclear (Jacobsen, 1973; Higgins et al., 1976; Mrva and Mares, 2001; Barrero et al., 2013; Zhang et al., 2022b). We observed that LT exposure upregulated six *α-amylase* genes in WTB seeds, as well as seven genes (including three homologous genes) related to GA biosynthesis/signaling and ABA catabolism (*TaGA20ox1*, *TaGID1-1B/1D*, *TaGASR7-7B/7D*, and *TaABA8′OH2-5B/5D*), and downregulated two α-amylase inhibitor genes (*TaCM1-7D* and *TaCM2-7B*) and two homologous genes related to ABA signaling (*TaABI5-3B/3D*). These transcriptional changes were accompanied by increased GA content and α-amylase activity, along with decreased ABA content in WTB seeds. In miR1832-overexpressing seeds, the expression of *α-amylase* genes, as well as genes related to GA biosynthesis/signaling and ABA catabolism, was downregulated, whereas expression of the two α-amylase inhibitor genes and the two ABA signaling genes was upregulated, leading to decreased GA_1_ and GA_3_ contents and α-amylase activity, increased ABA content, and enhanced SD. By contrast, *TaP450-7A*-overexpressing seeds exhibited the opposite trends. Together, these results suggest that the TaDof-2D–miR1832–TaP450-7A module mediates LT-induced dormancy release by coordinately regulating α-amylase activity and GA/ABA biosynthesis, catabolism, and signaling.

**Figure 7:**
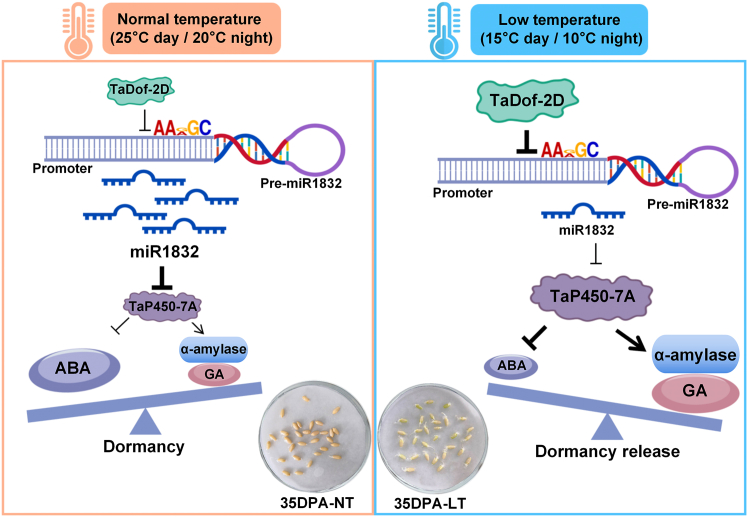
Molecular mechanism underlying low-temperature-induced seed dormancy release through modulation of α-amylase activity and the ABA and GA pathways. Exposure to low temperature (LT) during wheat seed development induces the expression of *TaDof-2D*. The transcription factor TaDof-2D directly binds to the dormancy-associated A allele of the miR1832 promoter and represses its transcription. This repression attenuates miR1832-mediated cleavage of the cytochrome P450 gene *TaP450-7A*. Elevated *TaP450-7A* expression subsequently alters the ABA/GA balance and α-amylase activity by upregulating genes involved in ABA catabolism (*TaABA8′OH-5B/5D*), GA biosynthesis (*TaGA20ox1*), and α-amylase production (*TaAmy1-6B*), while downregulating genes associated with ABA signaling (*TaABI5-3B/3D*) and α-amylase inhibition (*TaCM1-7D* and *TaCM2-7B*), ultimately leading to dormancy release. Blunt arrows (┴) indicate inhibition, and sharp arrows (→) indicate activation. Arrow thickness represents the relative strength of regulatory effects. NT, normal temperature.

We therefore propose a working model in which exposure to LT during seed development upregulates expression of the Dof TF TaDof-2D. As a transcriptional repressor, TaDof-2D binds directly to the dormancy-related A allele of the miR1832 promoter and inhibits its transcription. Reduced miR1832 levels relieve miR1832-mediated cleavage of the target gene *TaP450-7A*, thereby increasing *TaP450-7A* expression and promoting SD release and germination by modulating genes involved in GA and ABA pathways, the accumulation of GA and ABA, and α-amylase activity (Figure 7). Further investigation of the TaDof-2D–miR1832–TaP450-7A module will provide new insights into the molecular network underlying LT-regulated SD in wheat.

### Breeding potential of miR1832

Premature release of SD causes PHS, which has occurred more frequently in wheat and rice in recent years due to global climate change (Zhang et al., 2017; Mao et al., 2024). Therefore, improving PHS resistance has become an urgent goal in cereal breeding. We developed a CAPS marker (1832) based on the A/G mutation at −670 bp in the miR1832 promoter and verified its association with SD phenotypes using 160 WVs with different dormancy levels. Wheat varieties carrying the A allele exhibited stronger dormancy and lower PHS and therefore may serve as valuable genetic resources for improving PHS resistance in modern varieties. Notably, the favorable A allele was mainly found in landraces (76.6%) but rare in improved varieties (1.6%), indicating that this allele has not been fully utilized in modern wheat breeding. In addition, under field conditions, no obvious growth defects were observed in transgenic lines of *TaDof-2D*, miR1832, and *TaP450-7A*, nor in the EMS mutants of *TaDof-2D* and *TaP450-7A* (Supplemental Figure 14), implying their potential applicability in wheat breeding. Therefore, we propose that future breeding programs consider introducing the A allele into modern varieties to enhance dormancy levels and reduce the risk of PHS.

### Conclusion

In this study, we demonstrate that miR1832 plays a central role in LT-induced dormancy release in wheat. Under NT conditions, miR1832 positively regulates SD. The A allele at −670 bp in the miR1832 promoter is significantly associated with strong dormancy but is predominantly present in landraces and uncommon in improved varieties. We further identify the upstream TF TaDof-2D and the downstream target gene *TaP450-7A*, which together with miR1832 form the TaDof-2D–miR1832–TaP450-7A regulatory module. This module regulates SD under both LT and NT conditions through crosstalk with ABA and GA pathways. These results uncover a novel temperature-sensitive regulatory pathway and highlight the potential of miR1832 for PHS resistance breeding in wheat. By introducing the favorable A allele or editing key nodes within the TaDof-2D–miR1832–TaP450-7A pathway, wheat varieties with enhanced PHS resistance and improved adaptability to fluctuating climates may be developed. Notably, LT treatment during the middle and late stages of grain development can completely break dormancy, allowing harvested seeds to germinate immediately without stratification or other treatments, thereby improving breeding efficiency by shortening the dormancy–germination cycle.

## Methods

### Plant materials and growth conditions

The strong-dormancy wheat landrace WTB was cultivated under natural field conditions in Hefei (HF), China (31°58′N, 117°24′E) during the 2017–2018 cropping season (Supplemental Table 12). Experimental fields were maintained free of diseases and weeds. Wheat plants were exposed to either NT or LT prior to anthesis, followed by whole-transcriptome sequencing. Briefly, 60 representative spikes on the main stem were selected to mark flowering time, after which plants were transferred to two illuminated incubators (16 h day/8 h night, 70% relative humidity) for LT (10°C night/15°C day) or NT (20°C night/25°C day) treatment from 21 to 35 DPA. Seeds were collected at 21 DPA (21DPA-NT) and 35 DPA (35DPA-NT and 35DPA-LT), immediately frozen in liquid nitrogen, and stored at −80°C for RNA extraction.

Four wheat varieties, including WTB and HMC21 (strong dormancy) and ZY9507 and J411 (weak dormancy), were selected for miR1832 cloning and expression analysis. In total, 124 landraces and 245 improved varieties were grown during the 2021–2022 cropping season in HF (31°58′N, 117°24′E).

A total of 160 WVs exhibiting different SD levels were used to investigate the association between the A/G mutation (−670 bp) in the miR1832 promoter and the SD phenotype. All 160 WVs were grown during the 2021–2022 and 2022–2023 cropping seasons in HF (31°58′N, 117°24′E), Huaibei (HB; 33°54′N, 116°45′E), Suzhou (SZ; 33°29′N, 117°54′E), and Guohe (GH; 31°25′N, 117°9′E). The 160 WVs were mainly collected from the Chinese Huanghuai wheat region, the middle and lower Yangtze River wheat region, the northern wheat region, and the southwestern wheat region.

The WT wheat cultivar Fielder, miR1832 overexpression and STTM lines, *TaDof-2D* overexpression lines (in the Fielder background, carrying the G allele), and *TaP450-7A* overexpression lines were cultivated at the Dishang Experimental Station of the Hebei Academy of Agriculture and Forestry Sciences in Shijiazhuang, China (37°56′N, 114°43′E) during the 2023–2024 cropping season. Homozygous T_2_ transgenic plants were used in this study. The miR1832 and *TaP450-7A* overexpression hybrid lines (*pre-miR1832-OE#4/P450-7A-OE#7*), along with *TaDof-2D* overexpression lines carrying the A allele (*TaDof-2D-A-OE* lines, in the WTB background), were cultivated in a greenhouse maintained at 23°C ± 2°C under a 16 h light/8 h dark photoperiod. EMS mutants in the J411 background were used to validate the roles of *TaDof-2D* and *TaP450-7A* in SD, including *dof-2d* (2D_54677168_E) and *p450-7a* (7A_655307472_E). These mutants were provided by Boruidi Biotechnology Company (http://www.molbreeding.com). To minimize interference from background mutations, the mutants were backcrossed with J411 for two generations and then selfed to obtain BC_2_F_3_ progeny.

WT Columbia (Col-0) and transgenic *A. thaliana* lines (T_2_) were subjected to 3 days of cold treatment at 4°C, transferred to a greenhouse maintained at 24°C ± 1°C under a 16 h light/8 h dark photoperiod for 7 days, and subsequently transplanted into square pots filled with a 3:1 (v/v) mixture of vermiculite and black soil. WT Nipponbare (Nip; *Oryza sativa* L. ssp. *japonica*) and transgenic rice lines (T_2_) were grown in a greenhouse under the following conditions: 28°C day/25°C night; a 10 h light/14 h dark photoperiod; 70% relative humidity; and a light intensity of 200 μmol photons m^−2^ s^−1^.

### Vector construction and plant transformation

To construct the miR1832, *TaDof-2D*, and *TaP450-7A* overexpression vectors, a 250 bp stem–loop precursor sequence of miR1832 and the CDSs of *TaDof-2D* and *TaP450-7A* were amplified from the J411 genome and cloned into the pCAMBIA3301 vector under the control of the maize Ubi promoter (Liu et al., 2020). The STTM sequence for miR1832 was engineered according to established guidelines (Yan et al., 2012) and commercially synthesized by Youkang Biotech (Hangzhou, China). To generate the miR1832-STTM construct, the synthesized 90 bp miR1832-STTM fragment (CCGGCCGTCTAGCCGGCGACGGTTGTTGTTGTTATGGTCTAATTTAAATATGGTCTAAAGAAGAAGAATCCGGCCGTCTAGCCGGCGACG) was cloned into the pCAMBIA3301 vector. All recombinant constructs were introduced into *Agrobacterium tumefaciens* strain EHA105 and subsequently transformed into immature embryos of the wheat cultivar Fielder as described previously (Wang et al., 2017).

For Arabidopsis and rice transformation, the 250 bp stem–loop precursor sequence of miR1832 was amplified from the J411 and HMC21 genomes and cloned into the *Eco*RI and *Bam*HI sites of the pCAMBIA1300 binary vector. The miR1832-STTM fragment was then recombined into the pCAMBIA1300 expression vector. These constructs were introduced into *A. tumefaciens* strain GV3101 and used to transform Col-0 *A. thaliana* and Nip rice. Homozygous transgenic plants were confirmed by PCR analysis (Supplemental Table 13).

### Seed dormancy assay

The GI and GP were used to evaluate SD levels (Zuo et al., 2019). For wheat, 30 intact and healthy seeds were placed on filter paper moistened with 8 ml of distilled water in Petri dishes (90 mm diameter) and incubated at 22°C under a 14 h light/10 h dark photoperiod with 80% relative humidity. GI values for 160WVs were measured 5 days after harvest in 2022 (HF [2022GI5-HF], SZ [2022GI5-SZ], and HB [2022GI5-HB]) and 2023 (GH [2023GI5-GH], HF [2023GI5-HF], and HB [2023GI5-HB]) (Supplemental Table 14). For *A. thaliana*, seeds were placed on two layers of filter paper moistened with deionized water and incubated in a growth chamber at 23°C/21°C (day/night) under a 16 h light/8 h dark photoperiod. GP was scored on day 7, with radicle emergence defined as successful germination (Cao et al., 2020). For rice, seeds were placed on two layers of filter paper moistened with deionized water and incubated in a growth chamber set at 28°C/25°C (day/night) under a 10 h light/14 h dark photoperiod. GP was determined on day 7, and seeds were considered germinated when the radicle length was ≥1 mm (Li et al., 2020). For spike germination assays, at least five freshly harvested wheat spikes (35 DPA) were submerged in deionized water and vertically positioned in constant-temperature incubators at 20°C. Fresh water was replaced daily (Xu et al., 2022), and images were captured 5 days after treatment.

To measure seed moisture content, the sample box was weighed (±0.001 g) and recorded as M1. Seeds were then placed in the box, which was weighed and recorded as M2. The sample-filled box was dried in an oven at 100°C ± 5°C for 8 h, removed using crucible tongs, cooled to room temperature in a desiccator, and weighed and recorded as M3. Seed moisture content (%) was calculated as (M2 − M3)/(M2 − M1) × 100%, and values were rounded to two decimal places. Each sample consisted of three biological replicates.

### Cloning of miR1832 and development of a CAPS marker

The full-length miR1832 sequence was obtained from IWGSC RefSeq (v1.1). Primers were designed using Primer Premier (v5.0) and used to clone miR1832 from the genomes of J411 and HMC21 (Supplemental Table 13). PCR amplification was performed using 2× Phanta Max Master Mix (Vazyme, Nanjing, China) in a 20 μl reaction volume. PCR products were separated by 1.5% agarose gel electrophoresis, and the target fragments were excised, purified using an EasyPure Quick Gel Extraction Kit, cloned into the pEASY-Blunt Zero vector, and transformed into Trans-T1 chemically competent cells.

miR1832 sequence assembly and alignment using DNAMAN 6 revealed two sequence variants. A CAPS marker (1832) was developed using Primer Premier (v5.0) (Supplemental Table 13) based on a SNP (−670 bp, G/A) in the miR1832 promoter. The amplified 1832 fragment was digested with *Mnl*I (N_6_GAGG) for 3 h at 37°C, and the digestion products were resolved by 2.0% agarose gel electrophoresis.

### RNA extraction and sequencing library construction

Total RNA was extracted from nine samples representing 21DPA-NT, 35DPA-NT, and 35DPA-LT conditions, with three biological replicates per sample, using an RNA Extraction Kit (AG21017; Accurate Bio Inc., Hunan, China). Small-RNA sequencing libraries were constructed using the NEB Next Ultra Small RNA Sample Library Prep Kit (New England Biolabs, USA). Strand-specific mRNA and small-RNA sequencing were performed on an Illumina HiSeq 4000 platform at Biomarker Technologies (Beijing, China), following standardized Illumina protocols as described by Ma et al. (2015a).

### Transcriptomic analysis

For both mRNAs and lncRNAs, expression levels were calculated from RNA-seq data using the Expectation–Maximization algorithm implemented in RSEM (http://deweylab.biostat.wisc.edu/rsem). To enable comparisons among samples and eliminate the influence of gene length on read counts, fragments per kilobase of transcript per million mapped reads normalization was applied. Differential gene expression between groups was assessed based on fold change, and transcripts with a fold change ≥2.00 and a false discovery rate ≤0.01 were defined as DEGs (Wang et al., 2010). For small RNAs and circRNAs, expression levels were normalized as transcripts per million ( TPM; TPM = C × 10^6^/N) to allow inter-group comparisons and minimize the influence of sequencing depth on quantification accuracy.

DEGs were functionally annotated using the KEGG database (Ogata et al., 1999). TBtools (Chen et al., 2020) was used to identify overlapping differentially expressed RNAs among samples and tissues. Regulatory network diagrams were constructed using Cytoscape (v3.6.0; https://cytoscape.org). Target genes of differentially expressed miRNAs were predicted using the psRNATarget web server (http://plantgrn.noble.org/psRNATarget) (Dai and Zhao, 2011). Coding genes located within 100 kb upstream or downstream of lncRNAs were defined as *cis* targets.

### Real-time qPCR assay

Real-time qPCR was performed using the 2× SYBR Green Pro Taq HS Premix II quantitative PCR system (AG11702; Accurate Bio Inc., Hunan, China). First-strand cDNA synthesis for mRNAs and lncRNAs was performed using 0.5 μg of total RNA and the 5× Evo M-MLV RT Master Mix (AG11706; Accurate Bio Inc., Hunan, China) according to the manufacturer’s instructions. For circRNAs, RNase R (R0301; Jisai, China) was used to degrade linear RNAs prior to reverse transcription; first-strand cDNA synthesis was performed using random primers rather than oligo(dT) primers. Poly(A) tailing was used to enable miRNA quantification and was performed using an miRNA First-Strand Synthesis Kit (AG11716; Accurate Bio Inc., Hunan, China) following the manufacturer’s guidelines. *TaActin* was used as an internal reference gene, and three biological replicates were analyzed per assay. The wheat *U6* gene served as an internal control for miRNAs. Relative expression levels were calculated using the 2^−ΔΔCT^ method (Livak and Schmittgen, 2001). All RT-qPCR primer sequences are listed in Supplemental Table 13.

### Prediction and verification of miR1832 target genes

Targets of miR1832 were predicted using the psRNATarget program. A four-mismatch cutoff was applied to filter predicted miR1832 targets, with a G–U bond counted as a 0.5 mismatch. To identify cleavage sites within target mRNAs, RNA-ligase-mediated 5′-RACE was performed using a First Choice RLM-RACE Kit (AM1700; Thermo Fisher Scientific, USA) following the manufacturer’s protocol. For each RACE reaction, two gene-specific primers were used (Supplemental Table 13).

### Y1H screening and assay

Total RNA was extracted from HMC21 seeds collected at different infiltration periods and pooled to construct a full-length cDNA library. The cDNA was ligated into the linearized pGADT7-Rec vector to generate the library plasmid, which was subsequently transformed into *Saccharomyces cerevisiae* strain Y187 to construct the yeast activation domain (AD) library.

Because of the A/G SNP variation in the miR1832 promoter, 90-bp truncated promoter fragments containing either the A or G allele were generated. The fragment containing the A allele was cloned into the pAbAi vector (pAbAi-1832-A) and used as bait for library screening, whereas the fragment containing the G allele was cloned into pAbAi (pAbAi-1832-G) and used as a control. Recombinant vectors were transformed into chemically competent *S. cerevisiae* strain Gold1 cells and cultured on SD/−Ura medium at 30°C for 48 h. Bait-expressing strains were propagated on SD/−Ura medium, and serial dilutions of aureobasidin A (AbA) were added to determine the minimal inhibitory concentration (Supplemental Figure 15). The cDNA AD library plasmids were transformed into yeast strains harboring the pAbAi-1832-A vector and cultured on SD/−Leu medium supplemented with 200 ng/ml AbA. After 72 h, positive colonies were selected and re-cultured to eliminate false positives (Supplemental Figure 16). AD library plasmids were extracted from positive strains to identify binding transcripts. For one-to-one bait–prey interaction validation, the open reading frames of candidate binding transcripts were cloned into the pGADT7 vector and transformed into competent Gold1 yeast cells containing the pAbAi-1832-A vector. Transformed cells were cultured on SD/−Leu medium supplemented with 200 ng/ml AbA.

### EMSA

The full-length CDSs of *TaDof-2D*, *TaDof-3A*, and *TaDof-4D* were cloned in-frame downstream of GST in the pGEX-6P-1 vector. The fusion constructs were transformed into *Escherichia coli* BL21(DE3), and recombinant GST fusion proteins were purified using a Glutathione Sepharose 4 Fast Flow kit. For EMSA, single-stranded oligonucleotide probes were synthesized and annealed into double-stranded DNA by heating at 90°C–95°C in annealing buffer (10 mM Tris–HCl [pH 7.5], 1 mM EDTA, and 100 mM NaCl) for 5 min, followed by slow cooling to room temperature. Probe–protein interactions were analyzed following the protocol of the LightShift Chemiluminescent EMSA Kit (N20148; Thermo Fisher Scientific, USA).

### LUC reporter assay

Transient assays of miR1832–target interactions were performed using a dual-LUC reporter system (Geng et al., 2020). cDNAs encoding firefly LUC and *Renilla* LUC (REN) were individually cloned into a modified pCAMBIA1300 vector to generate the 35S:LUC and 35S:REN reporter constructs. The miR1832 precursor sequence was cloned into pCAMBIA1300 to generate the 35S:miR1832 expression vector. The *TaP450-7A* CDS was fused in-frame with the LUC reporter gene to generate the corresponding reporter plasmid. Leaves of 4-week-old *N. benthamiana* plants were infiltrated with *A. tumefaciens* carrying 35S:miR1832, the LUC reporter construct, and 35S:REN. Relative LUC activity (LUC/REN ratio) was determined 48 h post-infiltration using a dual-LUC reporter assay system on a multimode microplate reader (VICTOR Nivo, PerkinElmer, USA).

Transient transcriptional activity assays were performed in *N. benthamiana* leaves using the dual-LUC reporter system (Hellens et al., 2005). The miR1832 promoter was cloned into the pGreenII 0800-LUC reporter plasmid, and the CDSs of *TaDof-2D*, *TaDof-3A*, and *TaDof-4D* were inserted into the pGreenII 62-SK effector plasmid. Reporter and effector constructs were transformed into *A. tumefaciens* strain GV3101 and co-infiltrated into *N*. *benthamiana* leaves. The LUC/REN ratio was measured using a dual-LUC reporter assay system on a multimode microplate reader (VICTOR Nivo, PerkinElmer, USA).

### Measurement of GA and ABA contents and α-amylase activity

To measure ABA and GA contents and α-amylase activity, freshly harvested seeds were imbibed in a growth chamber (16 h light/8 h dark, 21°C ± 1°C) for 24 h prior to sampling. For ABA and GA quantification, imbibed seeds were frozen in liquid nitrogen, ground to a fine powder, and extracted with a mixed solution of isopropyl alcohol, hydrochloric acid, and water. An internal standard solution was added during sample preparation. Samples were quantified and identified by Wuhan ProNets Testing Technology Co., Ltd. (Wuhan, China) using high-performance liquid chromatography (Agilent 1290, USA) coupled with tandem mass spectrometry (AB SCIEX 6500 QTRAP, AB SCIEX, USA). Three biological replicates were analyzed per sample. α-amylase activity was determined using a commercial assay kit (BC0615; Beijing Solarbio Science & Technology Co., Ltd.) according to the manufacturer’s instructions.

### Statistical analysis

For GI values, descriptive statistical analyses and Mann–Whitney *U* tests were performed using SPSS 26.0 software (IBM, Armonk, NY, USA). Additional statistical analyses were conducted using GraphPad Prism (v8). Three biological replicates were used for each assay. Student’s *t*-tests were used to determine statistically significant differences between means at *∗P* < 0.05 and *∗∗P* < 0.01.

## Data and code availability

The raw sequence data generated in this study have been deposited in the National Center for Biotechnology Information (NCBI) under accession numbers PRJNA1412483 and PRJNA1409611, and in the China National Genomics Data Center (NGDC) under accession numbers CRA037244 and CRA037681. All other data supporting the findings of this study are available in the main text and the supplemental information.

## Funding

This work was supported by the 10.13039/501100001809National Natural Science Foundation of China (32372069; Joint Fund Projects, U20A2033), the 10.13039/501100018522Jiangsu Collaborative Innovation Center for Modern Crop Production (JCIC-MCP), and the Agricultural Research System of Anhui Province (AHCYTX-02).

## Acknowledgments

No conflict of interest is declared.

## Author contributions

W.G., C.C., and H.-p.Z. conceived and designed the experiments. W.G., Z.-h.C., H.X., J.-j.C., L.-t.Z., Y.-x.L., B.-b.T., and C.-x.H. performed the experiments. W.G., H.-p.Z., and Y.-L.R. wrote the manuscript. W.G., Z.-h.C., Z.-w.W., P.-b.H., J.L., C.C., C.-x.M., and H.-p.Z. participated in the experiments. W.G., Z.-h.C., C.C., and H.-p.Z. analyzed the data. All authors read and approved the final manuscript.

## Supplemental information

Document S1. Supplemental Figures 1–16 and Supplemental Tables 4, 8, 11, and 12

Supplemental Table 1. Differentially expressed mRNAs

Supplemental Table 2. Differentially expressed miRNAs

Supplemental Table 3. Differentially expressed lncRNAs

Supplemental Table 5. Comparison of expression data between RNA sequencing and RT-qPCR

Supplemental Table 6. RNAs mapped in the regulatory network

Supplemental Table 7. Genotypes of the CAPS marker 1832 detected in 160 wheat varieties.

Supplemental Table 9. Genotypes of the CAPS marker 1832 detected in 124 landraces and 245 improved varieties

Supplemental Table 10. Results of yeast one-hybrid screening of the pAbAi-miR1832-A library

Supplemental Table 13. Primers used in this study

Supplemental Table 14. Phenotypic data of seed dormancy in the 160 wheat varieties

Document S2. Article plus supplemental information
