# Supplementary material for: The TaDof-2D–miR1832–TaP450-7A module regulates low-temperature-induced seed dormancy release in wheat
Source: Plant Commun. 2026 Jan 30;7(7):101749. doi: 10.1016/j.xplc.2026.101749 (PMC13370231; doi:10.1016/j.xplc.2026.101749)
Supplement: Document S1. Supplemental Figures 1–16 and Supplemental Tables 4, 8, 11, and 12 [file mmc1.pdf]

Plant Communications, Volume 7

Supplemental information

The TaDof-2D–miR1832–TaP450-7A module regulates low-temperature-induced seed dormancy release in wheat

Wei Gao, Zi-heng Cui, Hua Xie, Jia-jia Cao, Li-tian Zhang, Yu-xia Lv, Bing-bing Tian, Chao-xu He, Zi-wei Wang, Pei-bo He, Jie Lu, Chuan-xi Ma, Cheng Chang, Yong-Ling Ruan, and Hai-ping Zhang

Supplemental Information

The TaDof-2D-miR1832-TaP450-7A module regulates low temperature-induced release of seed dormancy in wheat

Wei Gao[1, #], Zi-heng Cui[1, #], Hua Xie[1], Jia-jia Cao[1], Li-tian Zhang[1], Yu-xia Lv[1], Bing-bing Tian[1], Chao-xu He[1], Zi-wei Wang[1], Pei-bo He[1], Jie Lu[1], Chuan-xi Ma[1], Cheng Chang[1, *], Yong-Ling Ruan[2, 3, 4, *], Hai-ping Zhang[1, *]

[1]Key Laboratory of Wheat Biology and Genetic Improvement on Southern Yellow and Huai River Valley, College of Agronomy, Anhui Agricultural University, Hefei 230036, China.

[2]Innovation Cluster of Crop Molecular Biology and Breeding, Anhui Agricultural University, Hefei 230036, China.

[3]State Key Laboratory for Crop Stress Resistance and High-Efficiency Production and College of Horticulture, Northwest A&F University, Yangling 712100, China.

[4]Research School of Biology, The Australian National University, Canberra ACT 2601, Australia.

[#] These authors contributed equally to the work.

* Corresponding authors: Cheng Chang (changtgw@126.com); Yong-Ling Ruan (yong-ling.ruan@nwafu.edu.cn); Haiping Zhang (zhhp20@163.com).

Supplemental Figures

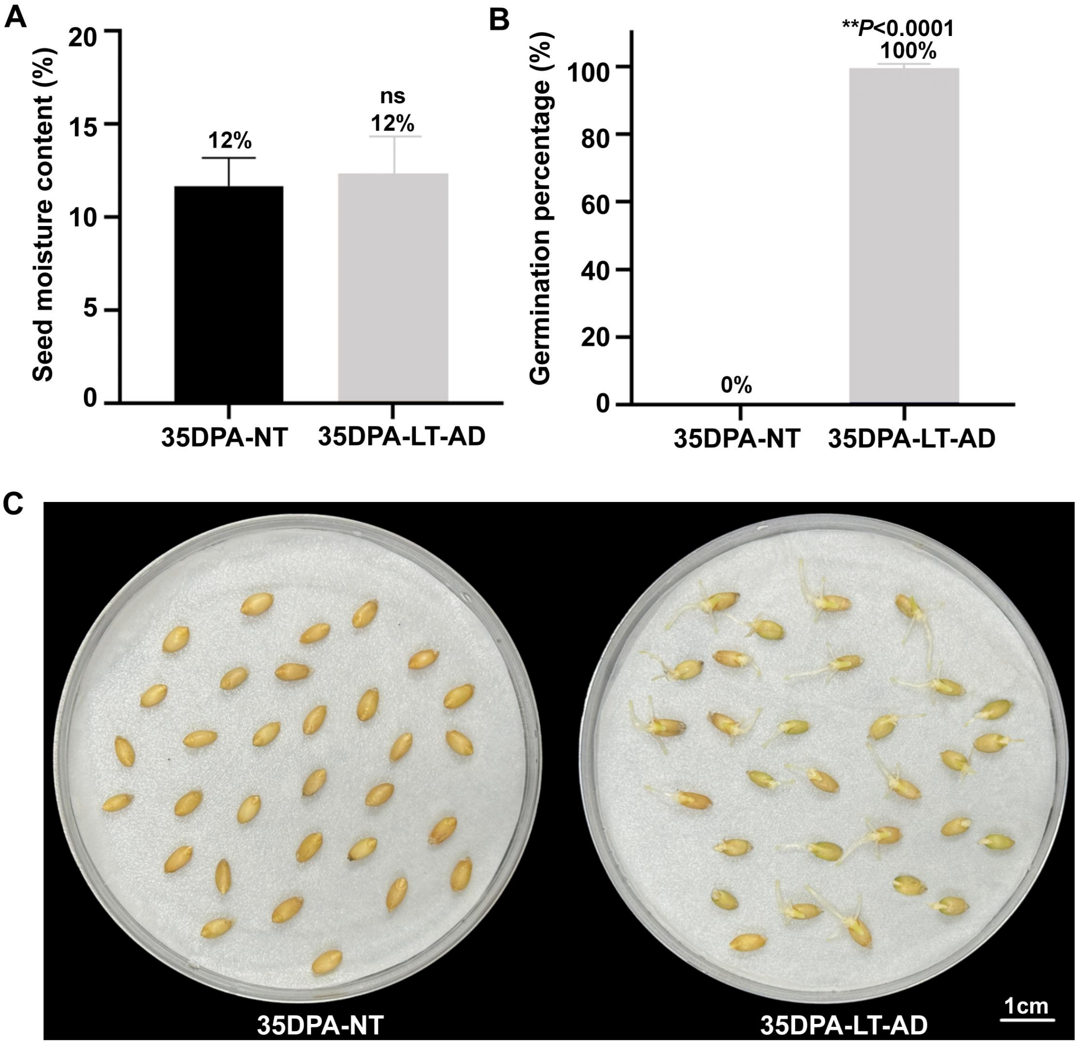

Supplemental Figure 1 Seed dormancy release is mediated by low temperature. (A) The moisture contents of NT-treated (35DPA-NT) and air-dried LT-treated (35DPA-LT-AD) 'WTB' seeds. The LT-treated seeds (23% moisture) were naturally air-dried until the moisture content dropped to 12%. **(B)** Germination percentages of 35DPA-NT and 35DPA-LT-AD seeds. Values are means ± SDs (n = 3). Statistical significance was analyzed using the Student's *t*-test (**P* < 0.05 and ***P* < 0.01). ns, not significant. **(C)** Germination phenotypes of 35DPA-NT and 35DPA-LT-AD seeds imbibed for three days. AD, air-dried.

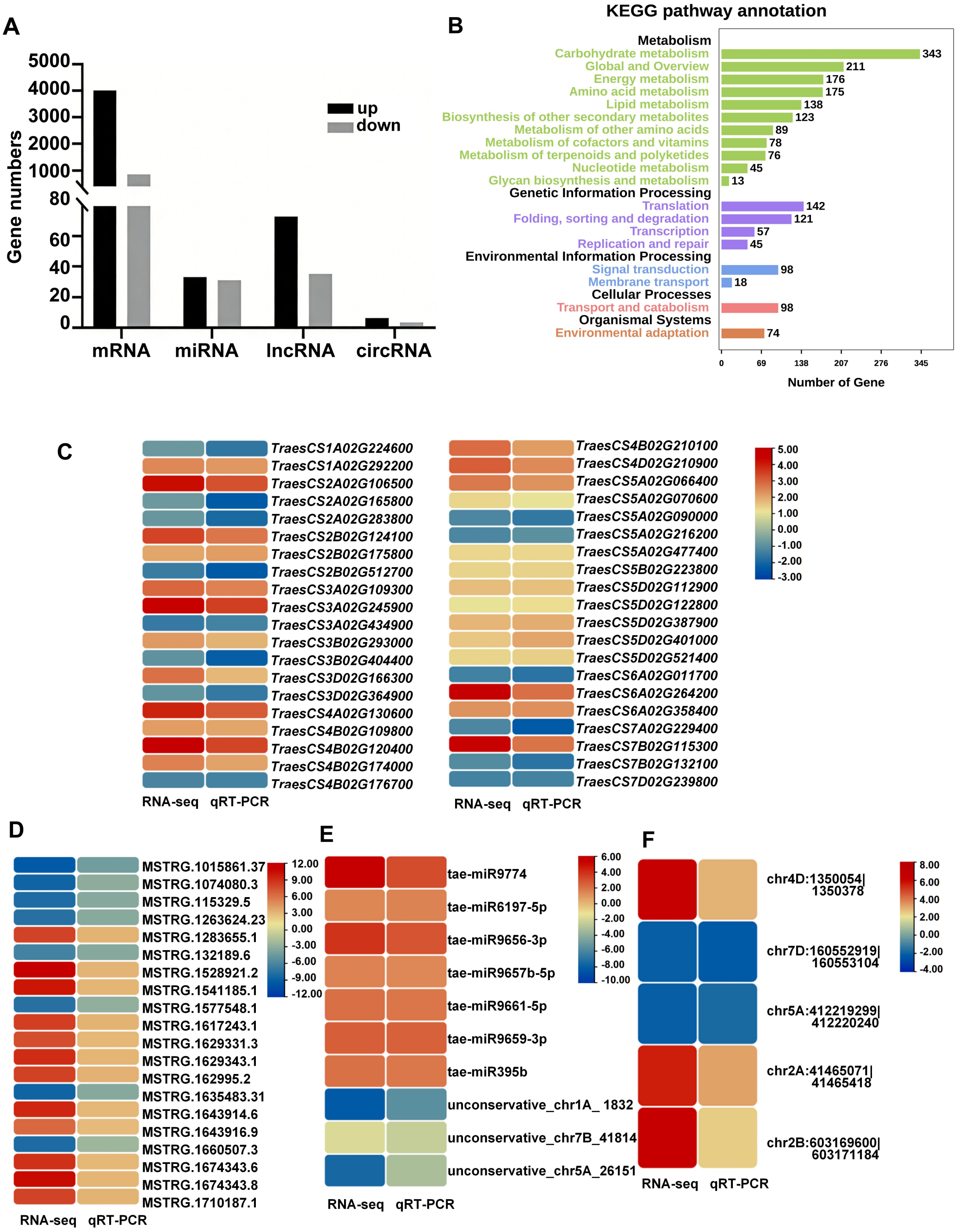

Supplemental Figure 2 Identification, functional analysis and expression validation of differentially expressed mRNAs, miRNAs, lncRNAs and circRNAs in wheat seeds under 35DPA-NTvsLT conditions. (A) Differentially expressed RNAs between ‘WTB’ wheat seeds collected at 35 days post anthesis under low temperature (LT) and normal temperature (NT) conditions (35DPA-NTvsLT). **(B)** KEGG pathway analysis of differentially expressed mRNAs identified in 35DPA-NTvsLT. **(C)** Expression patterns of the selected mRNAs detected by RNA-seq and qRT-PCR. **(D)** Expression patterns of the selected lncRNAs detected by RNA-seq

and qRT-PCR. **(E)** Expression patterns of the selected miRNAs detected by RNA-seq and qRT-PCR. **(F)** Expression patterns of the selected circRNAs detected by RNA-seq and qRT-PCR.

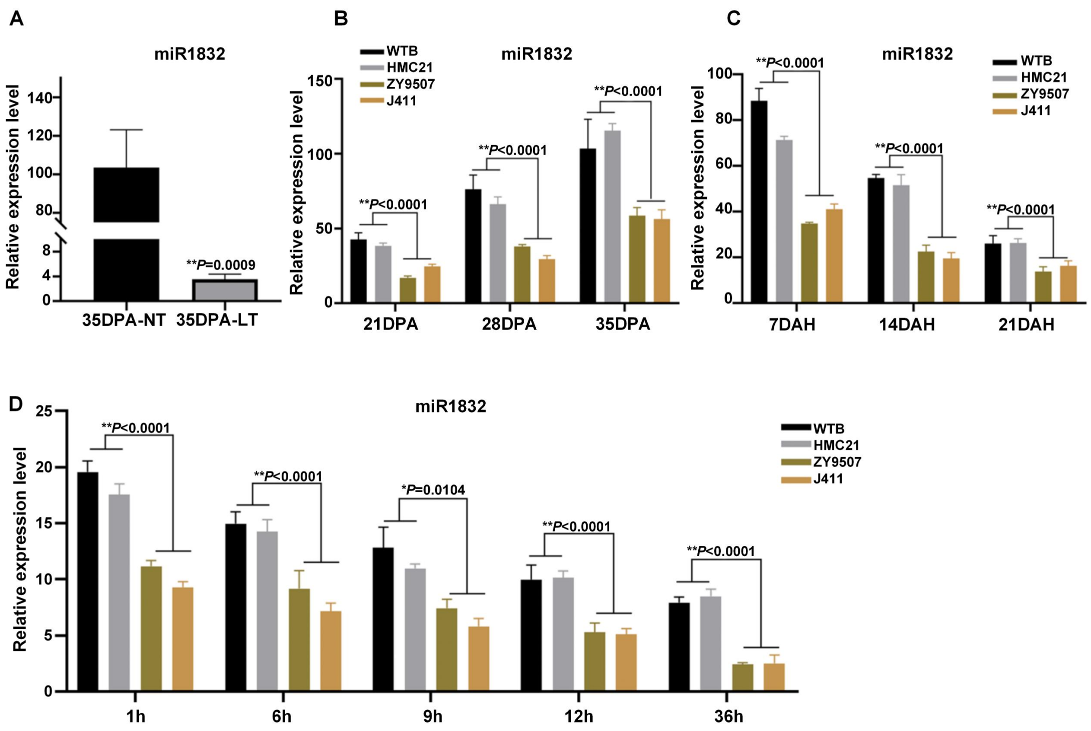

Supplemental Figure 3 Expressions analysis of miR1832 at different stages. (A) Expression levels of miR1832 in Waitoubai (WTB) seeds treated with low temperature by qRT-PCR. Values are means ± SDs (n = 3). **(B)** Expression patterns of miR1832 at different developmental stages (21, 28, and 35 days post anthesis [DPA]) by qRT-PCR. **(C)** Expression profiles of miR1832 at different post-ripening stages (7, 14, and 21 days after harvest [DAH]) by qRT-PCR. **(D)** Expression profiles of miR1832 at different imbibition stages in WTB, HMC21, ZY9507 and J411 seeds. Values are means ± SDs (n = 3). Statistical significance was analyzed using the Student's *t*-test (*$P < 0.05$ and **$P < 0.01$).

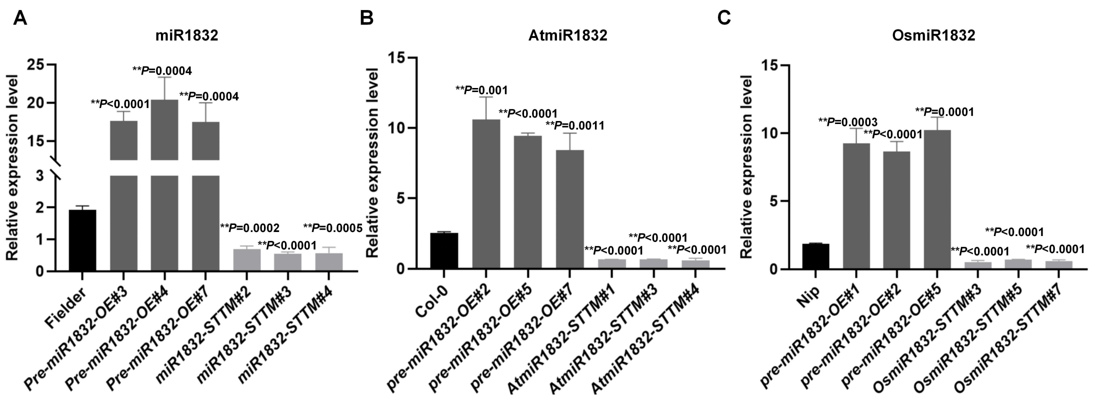

Supplemental Figure 4 Expressions analysis of miR1832, AtmiR1832, and OsmiR1832. (A) Expression levels of miR1832 in wheat overexpression lines *pre-miR1832OE#3/4/7*, STTM lines *miR1832-STTM#2/3/4*, and wild-type Fielder by qRT-PCR. Values are means ± SDs (n = 3). **(B)** Expression levels of AtmiR1832 in Arabidopsis overexpression lines *pre-miR1832-OE#2/5/7*, STTM lines *AtmiR1832-STTM#1/3/4*, and wild-type Col-0 by qRT-PCR. Values are means ± SDs (n = 3). **(C)** Expression levels of OsmiR1832 in rice overexpression lines *pre-miR1832-OE#1/2/5*, STTM lines *OsmiR1832-STTM#3/5/7*, and wild-type Nip by qRT-PCR. Values are means ± SDs (n = 3). Statistical significance was analyzed using the Student's *t*-test (*P < 0.05 and **P < 0.01).

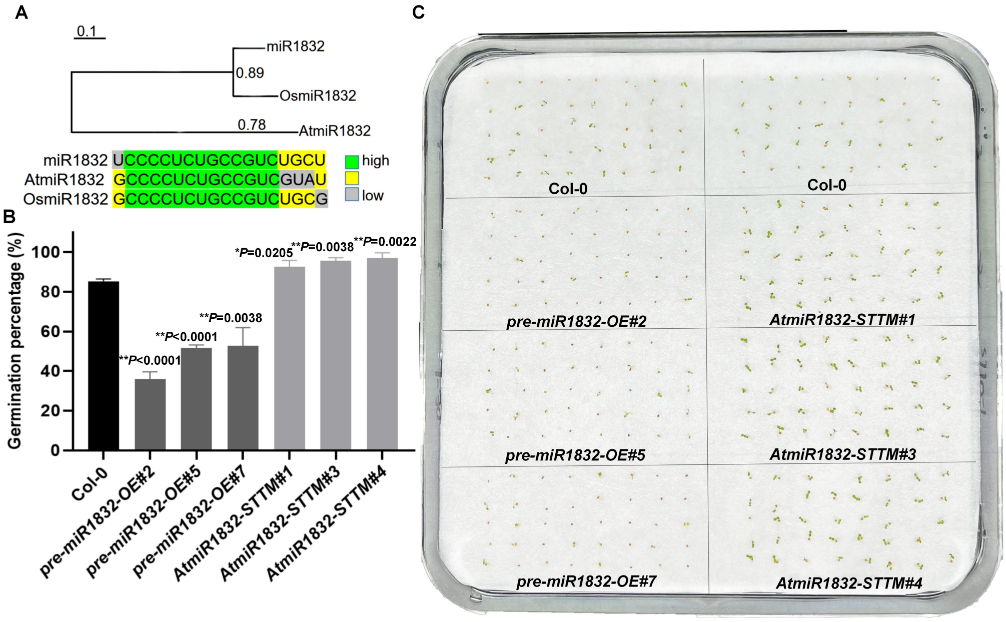

Supplemental Figure 5 miR1832 positively regulates Arabidopsis seed dormancy. (A) Phylogenic tree analysis of wheat miR1832, rice OsmiR1832, and Arabidopsis AtmiR1832. **(B)** Germination percentages of miR1832 overexpression lines *pre-miR1832-OE#2/5/7*, STTM lines

AtmiR1832-STTM#1/3/4, and wild-type Col-0 seeds imbibed for 7 days. Values are means ± SDs (n = 3). Statistical significance was analyzed using the Student's *t*-test (**P* < 0.05 and ***P* < 0.01). **(C)** Germination images of *pre-miR1832-OE#2/5/7*, *AtmiR1832-STTM#1/3/4*, and Col-0 seeds imbibed for7 days.

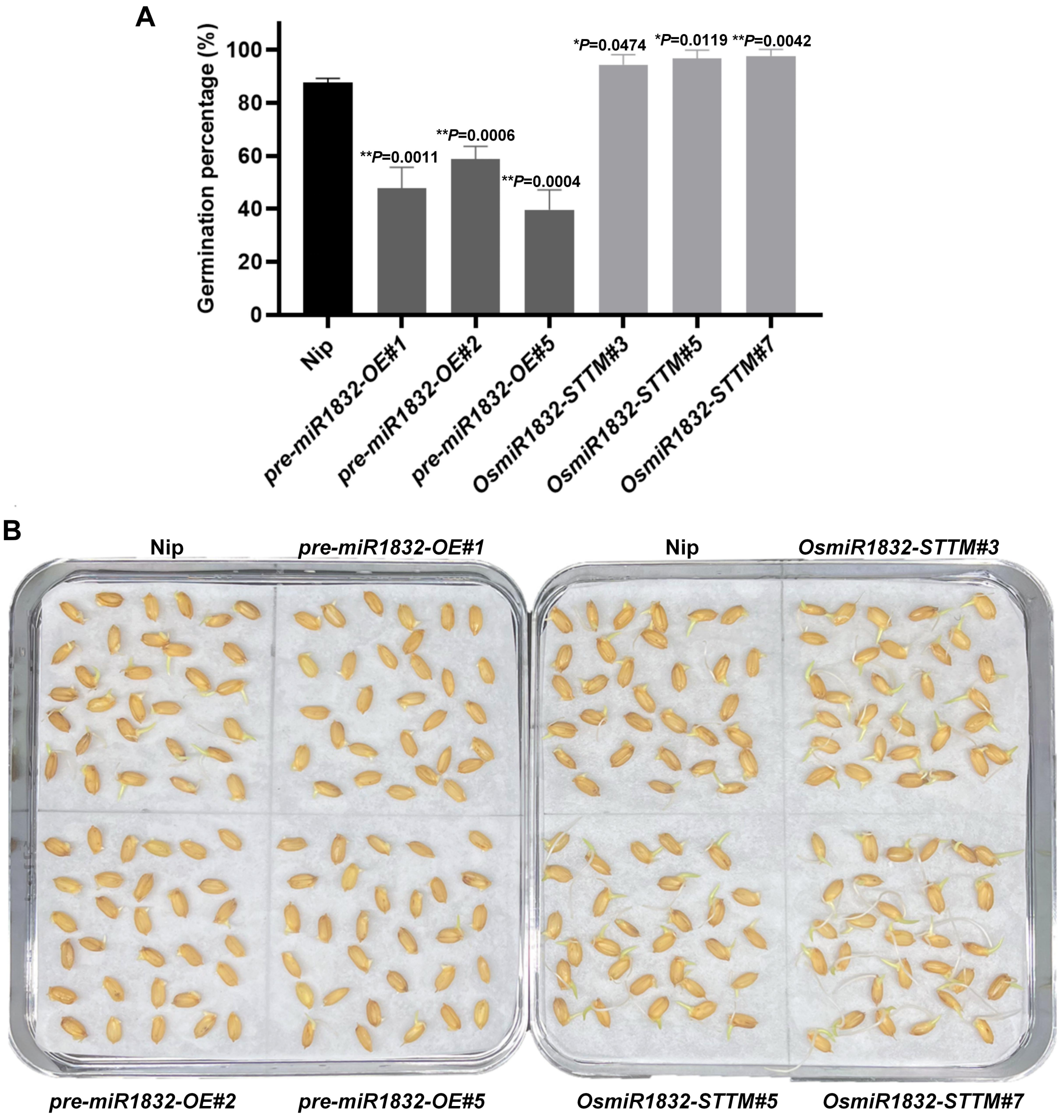

Supplemental Figure 6 miR1832 positively regulates rice seed dormancy. (A) Germination percentages of miR1832 overexpression lines *pre-miR1832-OE#1/2/5*, STTM lines *OsmiR1832-STTM#3/5/7*, and wild-type Nip seeds imbibed for 7 days. Values are means ± SDs (n = 3). Statistical significance was analyzed using the Student's *t*-test (**P* < 0.05 and ***P* < 0.01). **(B)** Germination images of *pre-miR1832-OE#1/2/5*, *OsmiR1832-STTM#3/5/7*, and Nip seeds imbibed for 7 days.

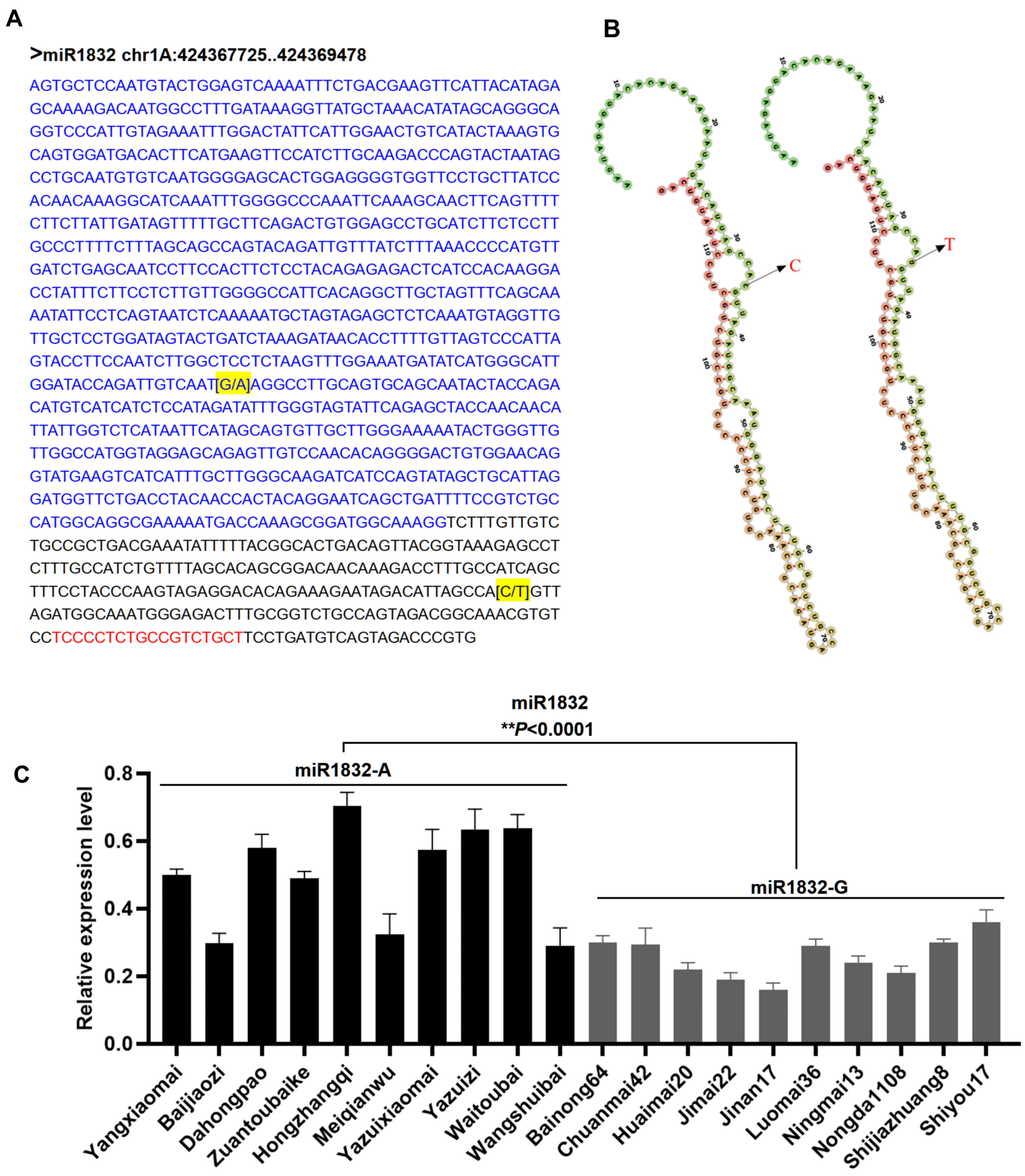

Supplemental Figure 7 Sequence and expression analysis of miR1832. (A) DNA sequences comparison of miR1832 between the weak-dormancy varieties (ZY9507 and J411) and the strong-dormancy varieties (WTB and HMC21). miR1832 promoter (blue), precursor (black, mature sequence in red), and mutation loci (yellow-highlighted). **(B)** The stem-loop structure analysis of miR1832. **(C)** The expression of miR1832 in wheat varieties carrying the A or G allele. Values are means ± SDs (n = 3). Statistical significance was analyzed using the Student's *t*-test (**$P < 0.01$).

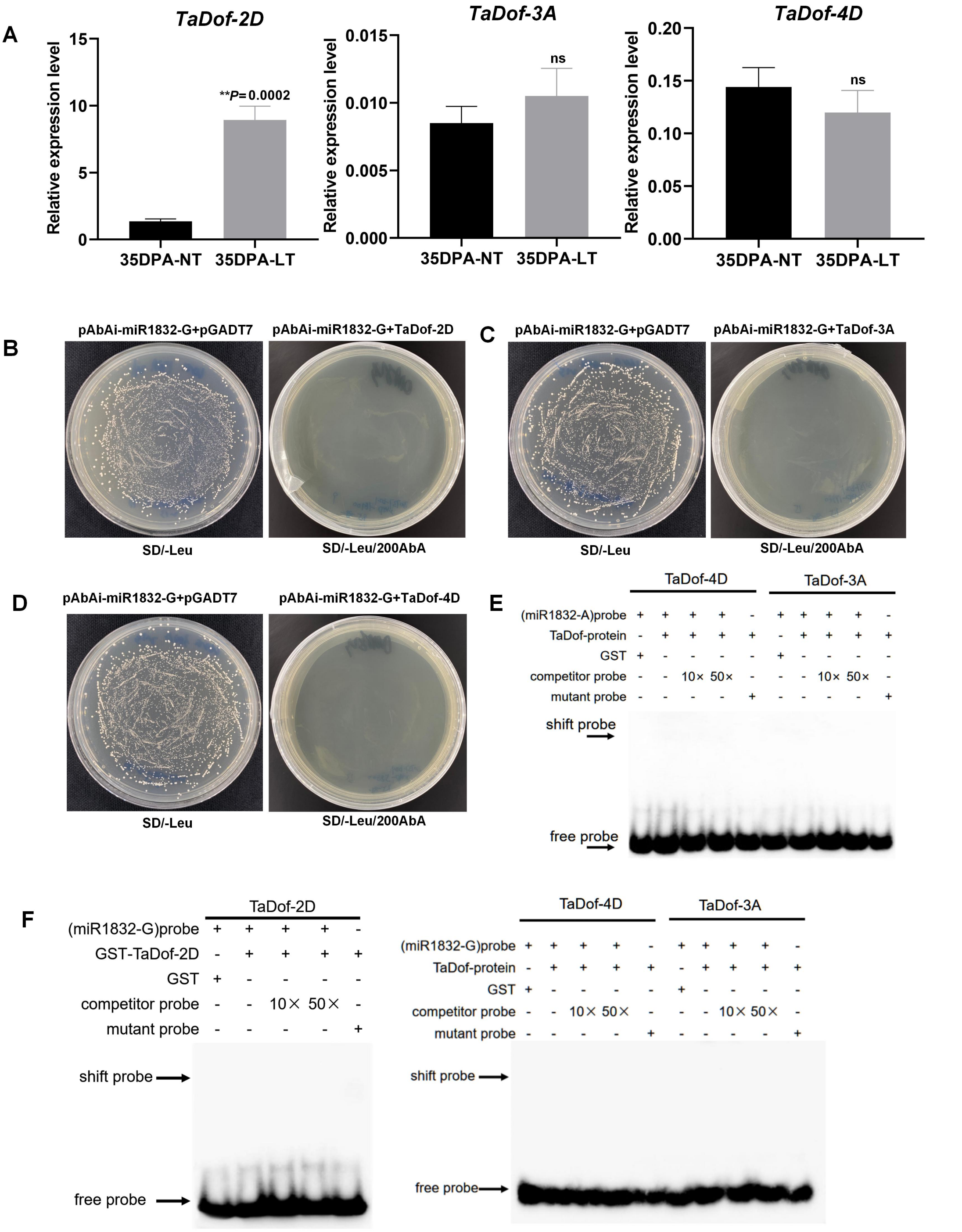

Supplemental Figure 8 TaDof-2D directly binds to the miR1832 promoter containing the A allele. (A) Expression levels of *TaDof-2D*, *TaDof-3A* and *TaDof-4D* in WTB seeds treated with low temperature by qRT-PCR. Values are means ± SDs (n = 3). Statistical significance was analyzed using the Student's *t*-test (***P* < 0.01). ns, not significant. **(B-D)** Yeast one-hybrid assay (Y1H) showing that TaDof-2D/3A/4D do not bind to the miR1832 promoter containing the G allele. **(E)** Electrophoretic mobility shift assay (EMSA) was performed to evaluate the ability of

TaDof-3A/4D to bind to the Dof motif AAGGC in the miR1832 promoter region. **(F)** Electrophoretic mobility shift assay (EMSA) was performed to evaluate the ability of TaDof-2D/3A/4D to bind to the Dof motif GAGGC in the miR1832 promoter region. Ten-fold and 50-fold unlabeled probes are added as competitors in the binding reaction.

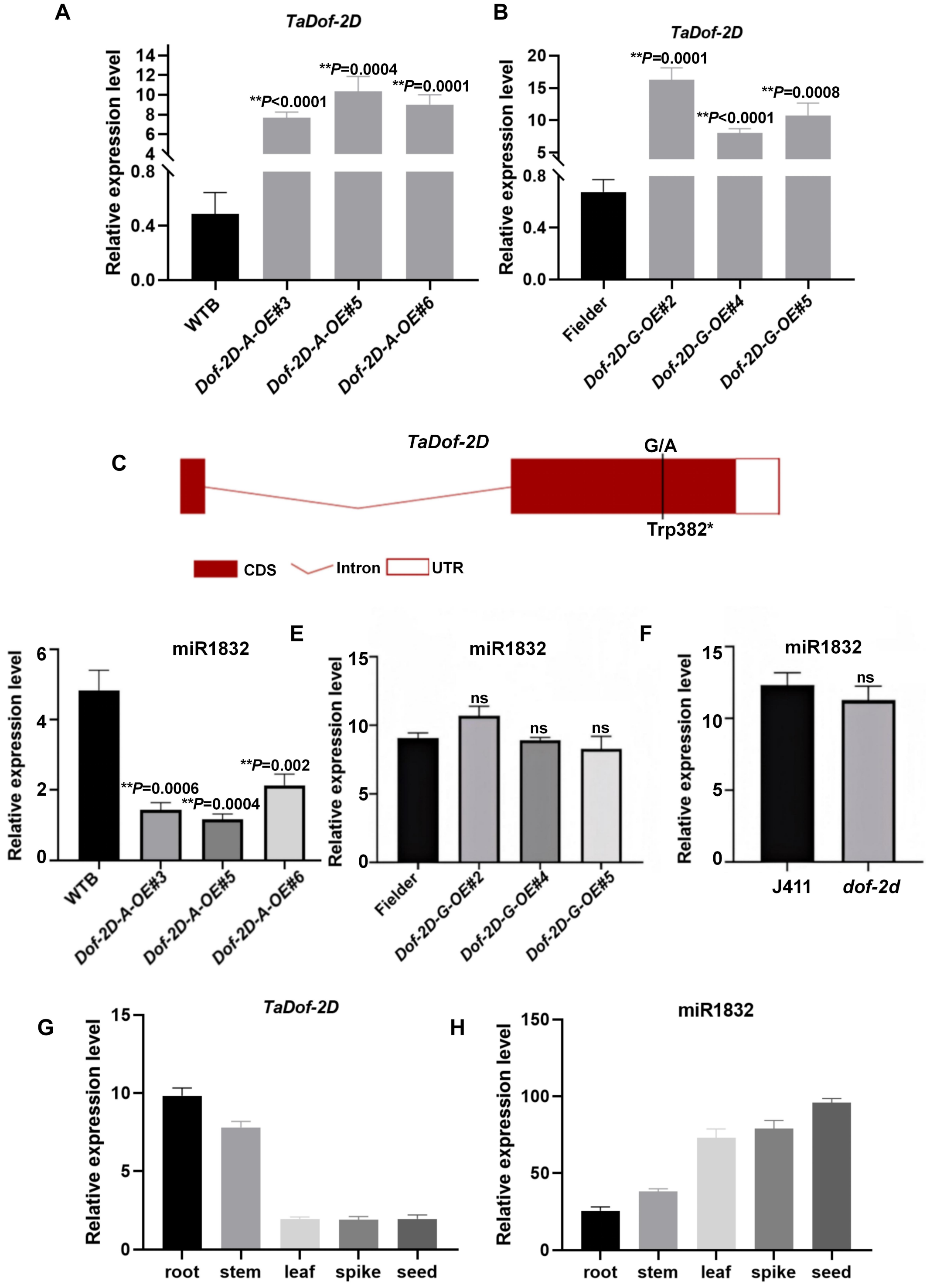

Supplemental Figure 9 Expression analysis of *TaDof-2D* and miR1832. (A) The expression of *TaDof-2D* in *Dof-2D-A-OE* and wild-type 'WTB' seeds by qRT-PCR. Values are means ± SDs (n = 3). **(B)** The expression of *TaDof-2D* in *Dof-2D-G-OE* and wild-type Fielder seeds by qRT-PCR. Values are means ± SDs (n = 3). **(C)** Mutation site of the wheat EMS mutant *dof-2d*. **(D)** The expression of miR1832 in *Dof-2D-A-OE* and wild-type WTB seeds by qRT-PCR. Values are means ± SDs (n = 3). **(E)** The expression of miR1832 in *Dof-2D-G-OE* and wild-type Fielder seeds by qRT-PCR. Values are means ± SDs (n = 3). **(F)** The expression of miR1832 in *dof-2d* seeds by qRT-PCR. Values are means ± SDs (n = 3). Statistical significance was analyzed using the Student's *t*-test (**$P < 0.01$). ns, not significant. **(G-H)** The expression patterns of *TaDof-2D* and miR1832 in roots, stems, leaves, spikes, and seeds of 'WTB'.

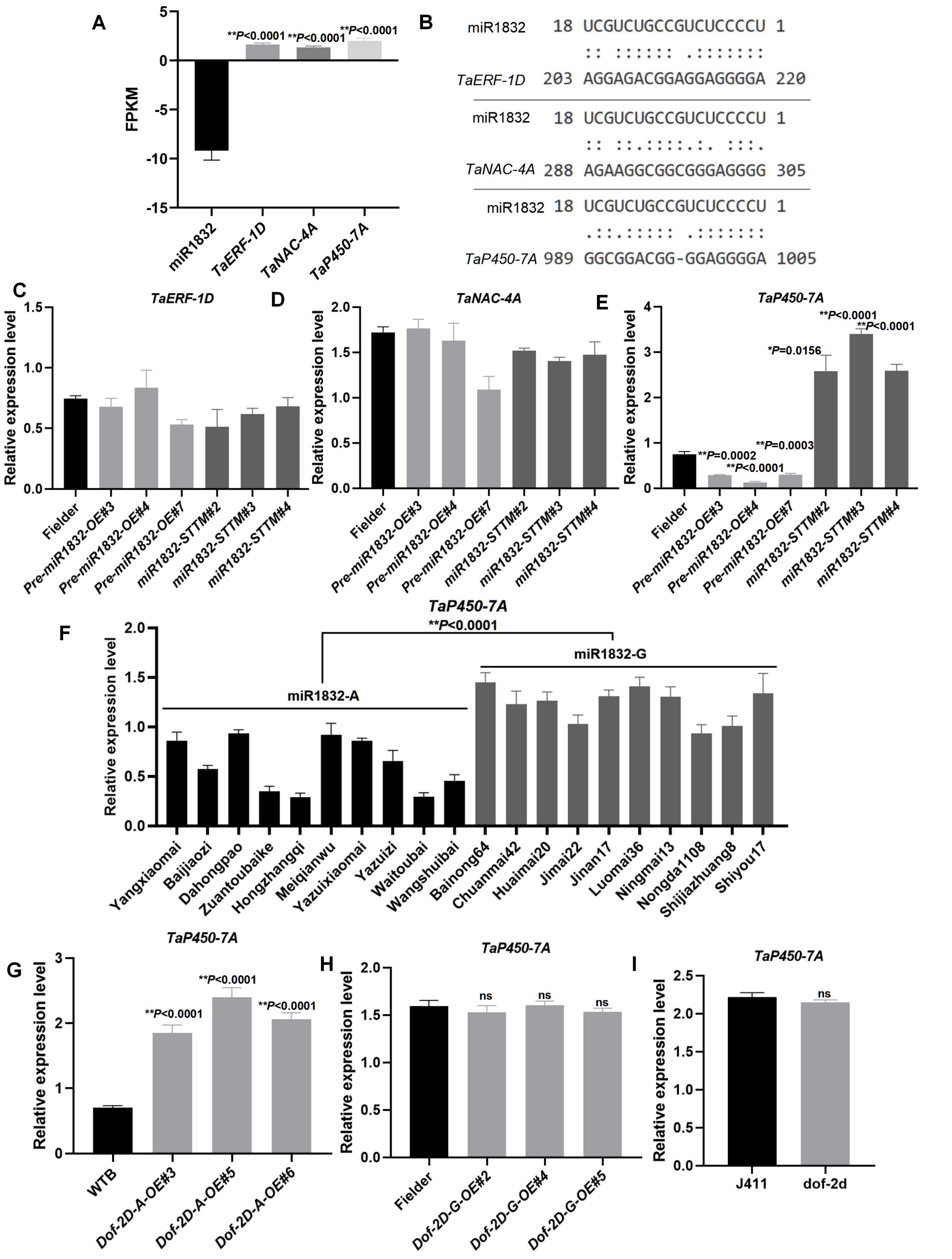

Supplemental Figure 10 Identification of miR1832 target genes. (A-B) Expression and sequence pairing of miR1832 and candidate target genes (*TaERF-1D*, *TaNAC-4A*, and *TaP450-7A*) in 35DPA-NTvsLT. **(C-E)** Expression analysis of *TaERF-1D*, *TaNAC-4A*, and *TaP450-7A* in *pre-miR1832-OE*, *miR1832-STTM*, and wild-type Fielder. Values are means ± SDs (n = 3). **(F)** The expression of *TaP450-7A* in wheat varieties carrying the A (miR1832-A) or G (miR1832-G) allele in the miR1832 promoter. Values are means ± SDs (n = 3). **(G)** The expression of *TaP450-7A* in *Dof-2D-A-OE* and wild-type WTB seeds by qRT-PCR. Values are means ± SDs (n = 3). **(H)** The

expression of *TaP450-7A* in *Dof-2D-G-OE* and wild-type Fielder seeds by qRT-PCR. Values are means ± SDs (n = 3). **(I)** The expression of *TaP450-7A* in *dof-2d* seeds by qRT-PCR. Values are means ± SDs (n = 3). Statistical significance was analyzed using the Student's *t*-test (**P* < 0.05 and ***P* < 0.01).

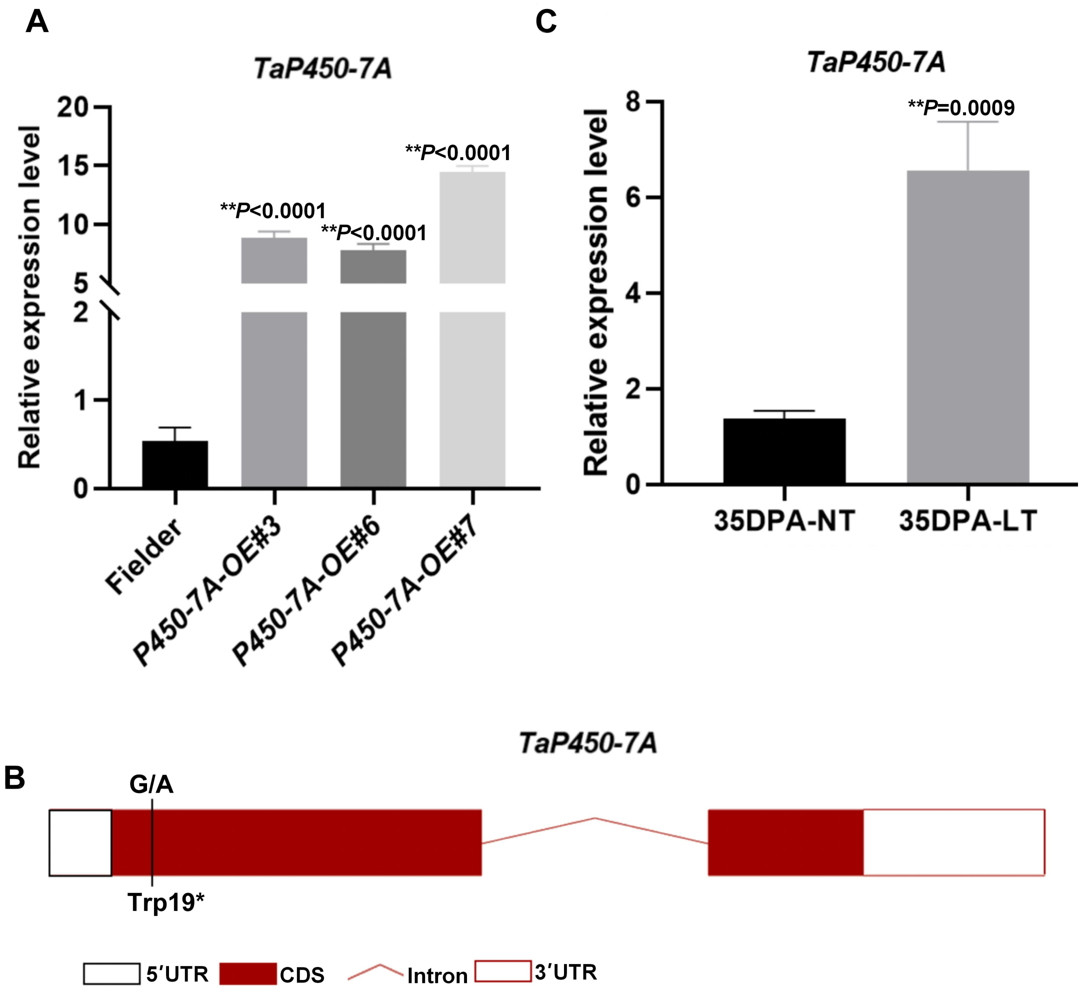

Supplemental Figure 11 Expression analysis of *TaP450-7A* and the mutation site of wheat EMS mutant *p450-7a*. **(A)** Expression levels of *TaP450-7A* in overexpression lines *P450-7A-OE#3/6/7* and wild-type Fielder by qRT-PCR. Values are means ± SDs (n = 3). **(B)** Mutation site of the wheat EMS mutant *p450-7a*. **(C)** Expression levels of *TaP450-7A* in WTB seeds treated with low temperature by qRT-PCR. Values are means ± SDs (n = 3). Statistical significance was analyzed using the Student's *t*-test (***P* < 0.01).

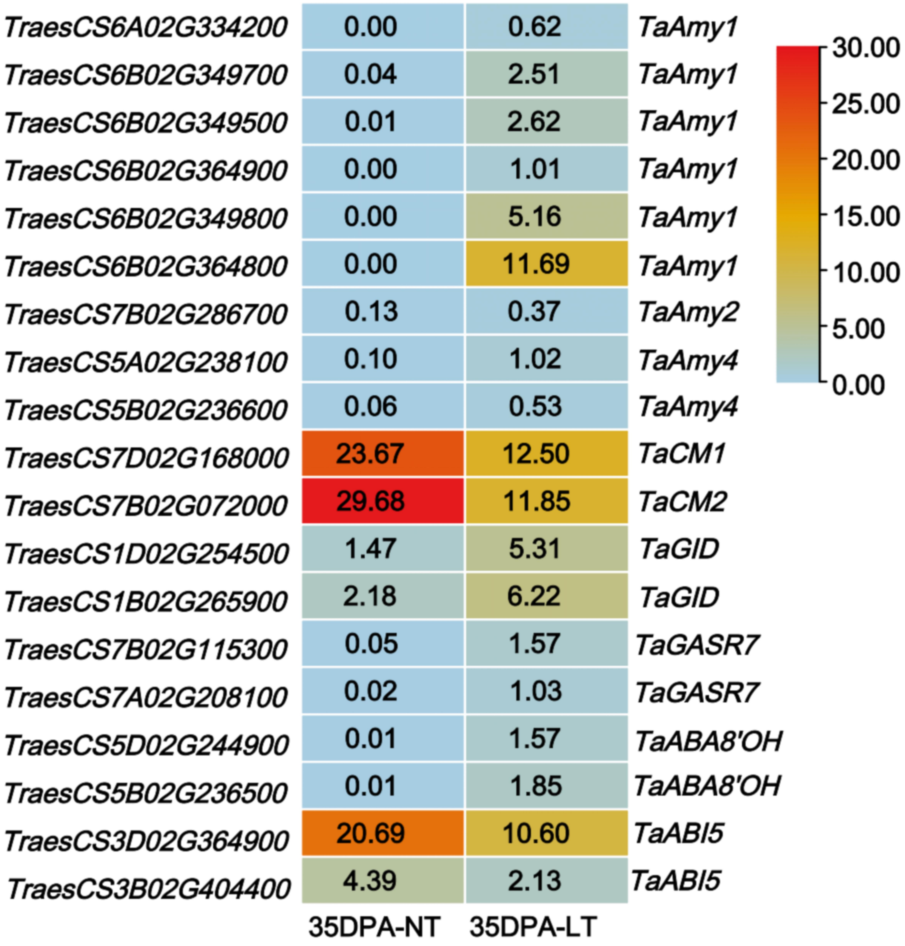

Supplemental Figure 12 Expression levels of genes involved in GA and ABA signaling pathways in WTB seeds treated with low temperature and normal temperature at 35 days post anthesis (35DPA-LT and 35DPA-NT).

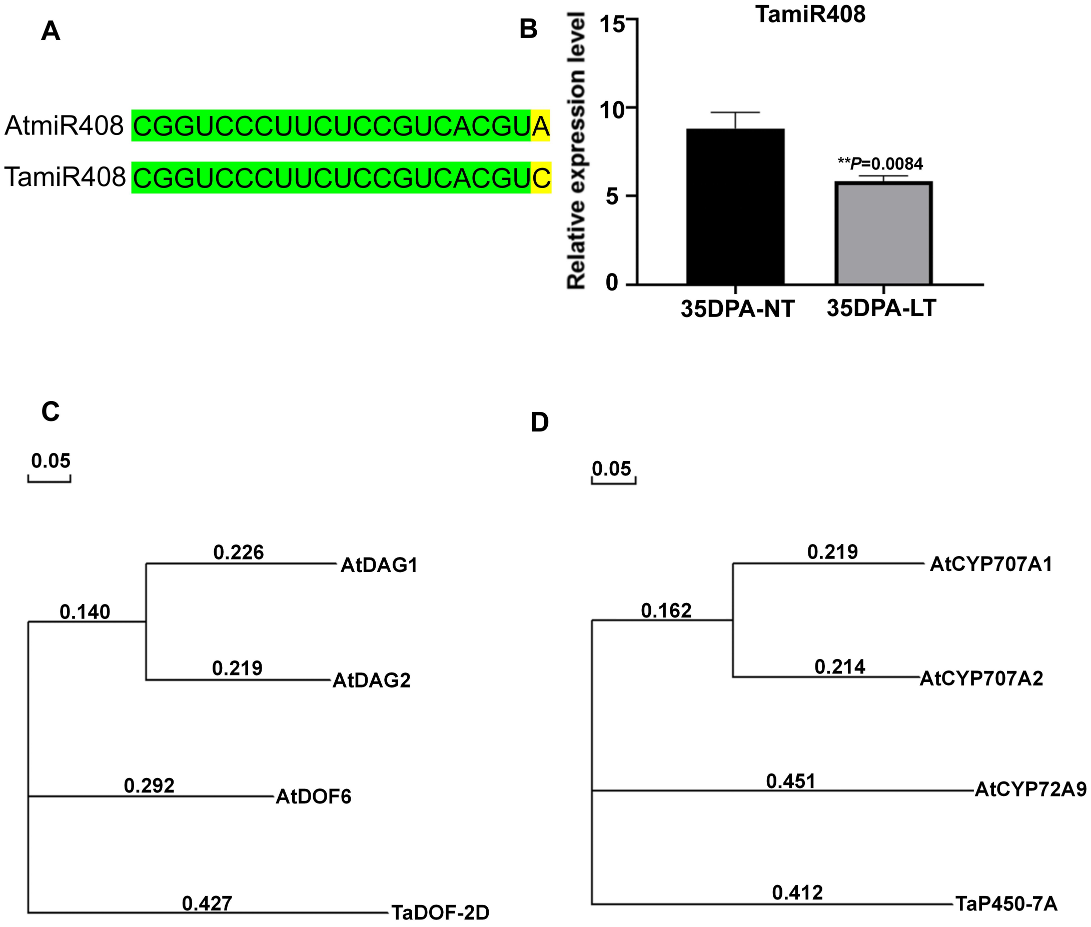

Figure S13 Sequence and expression analysis of miR408 and phylogenetic tree analysis of TaDof-2D and TaP450-7A. (A) Sequence alignment of miR408 in wheat and Arabidopsis. **(B)** Relative expression of miR1832 in Waitoubai (WTB) seeds treated with low temperature by qRT-PCR. Values are means ± SDs (n = 3). Statistical significance was analyzed using the Student's *t*-test (***P* < 0.01). **(C-D)** Homologous phylogenetic tree of the TaDOF-2D and TaP450-7A protein. MEGA v.7.0 was used to produce the neighbor-joining tree with 1000 bootstrap replicates. Branch length represents the evolutionary distance. The shorter the branch length, the smaller the difference and the closer the evolutionary distance. Initial letters indicate diferent plants: Ta (*Triticum aestivum*) and At (*Arabidopsis thaliana*).

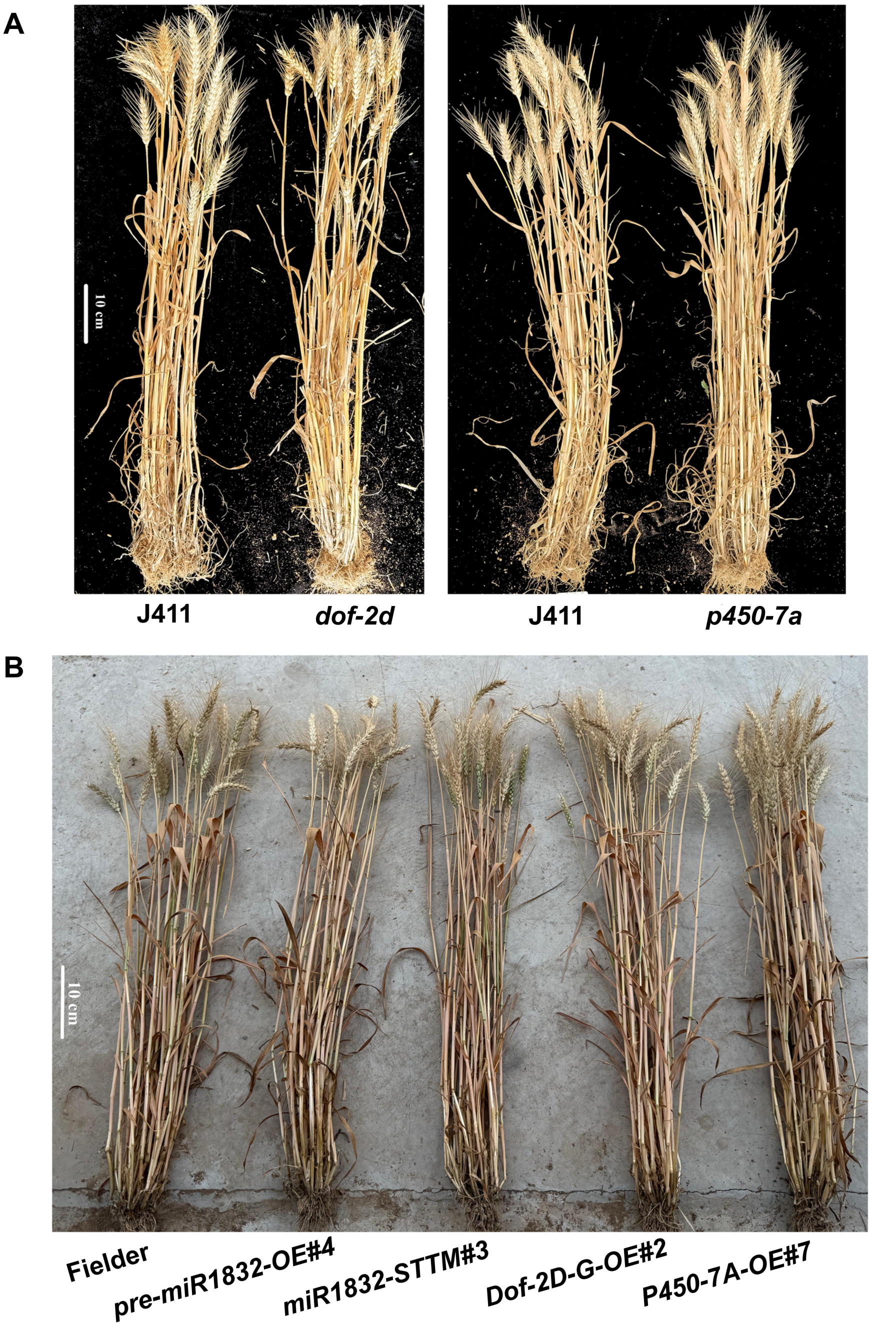

Supplemental Figure 14 Plant images of miR1832, *TaDof-2D*, and *TaP450-7A* transgenic lines and EMS mutants. (A) Plant images of *dof-2d* (*TaDof-2D* EMS mutant), *p450-7a* (*TaP450-7A* EMS mutant), and wild-type J411 in the field. **(B)** Plant images of *pre-miR1832-OE#4*, *miR1832-STTM#3*, *Dof-2D-G-OE#2*, *P450-7A-OE#7*, and wild-type Fielder in the field. Scale bar = 10 cm.

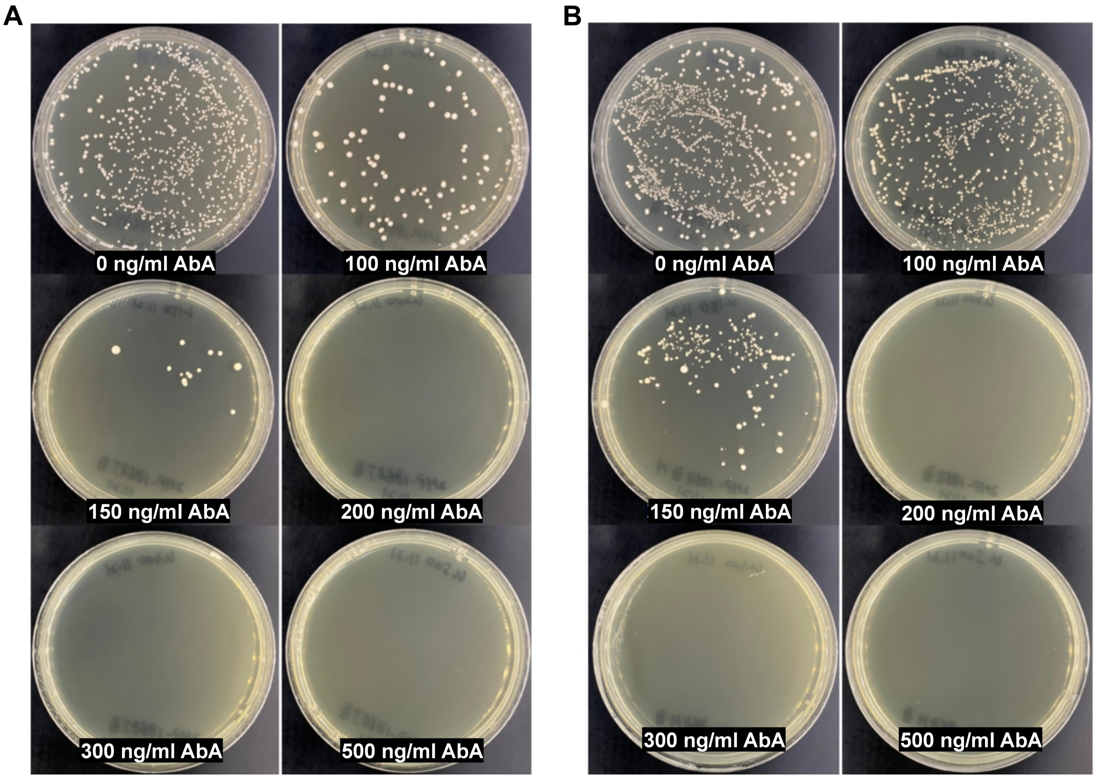

Supplemental Figure 15 Self-activation verification of yeast strain Gold1. (A) Self-activation verification of pAbAi-1832-G at different AbA concentrations (0-500 ng/ml AbA). **(B)** Self-activation verification of pAbAi-1832-A at different AbA concentrations (0-500 ng/ml AbA).

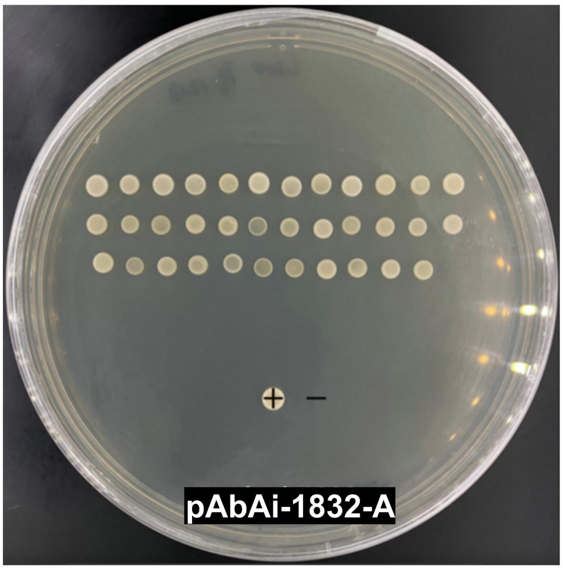

Supplemental Figure 16 Secondary screening of yeast strain Gold1. Secondary screening of yeast strain pAbAi-1832-A at a Concentration of 200 ng/ml AbA.

Supplemental Tables

Supplemental Table 4 Differentially expressed circRNAs.

Number	circRNA ID	log2Fold Change 35DPA-NTvsLT	regulated	Origin gene
1	chr2A:41465071|41465418	5.62	up	Wheat_newGene_1136962_1
2	chr2B:603169600|603171184	6.22	up	Wheat_newGene_1329988_Wheat_newGene_1329989_1
3	chr4B:69932527|69933411	5.80	up	Wheat_newGene_17545_Wheat_newGene_17546_1
4	chr4B:81931658|81933633	6.05	up	Wheat_newGene_19389_2
5	chr4D:1350054|1350378	6.36	up	TraesCS4D02G002800_1
6	chr5A:412219299|412220240	-2.12	down	Wheat_newGene_252882_Wheat_newGene_252883_1
7	chr6B:231687840|231692714	2.58	up	Wheat_newGene_596613_Wheat_newGene_596612_1
8	chr7D:160552919|160553104	-2.13	down	TraesCS7D02G201500_2
9	chrUn:109025131|109025506	-6.09	down	TraesCSU02G127100_1

Supplemental Table 8 Descriptive statistics and Mann-Whitney U test results of seed germination index (GI) values between the two alleles (1832-G and 18332-A) of 1832 in 160 wheat varieties (160WVs).

Trait	*1832-G* Mean±SD	*1832-A* Mean±SD	*U-test*
2022GI5-HF	0.23±0.19	0.06±0.11	4.893**
2022GI5-SZ	0.45±0.28	0.30±0.24	2.613**
2022GI5-HB	0.40±0.26	0.25±0.26	2.575*
2023GI5-GH	0.71±0.29	0.17±0.21	6.550**
2023GI5-HF	0.67±0.31	0.17±0.21	6.401**
2023GI5-HB	0.73±0.32	0.32±0.38	4.489**

Supplemental Table 11 Expression data of known genes involved in GA and ABA signaling pathways in 35DPA-NTvsLT.

RNA ID	log2Fold Change (35DPA-NTvsLT)				NR annotation
	RNA-seq	regulated	qRT-PCR	regulated	
TraesCS6A02G334200	9.58	up	4.27	up	alpha-amylase 1 [Triticum aestivum] 6A
TraesCS6B02G349700	5.56	up	4.05	up	alpha-amylase 1 [Triticum aestivum] 6B
TraesCS6B02G349500	7.71	up	4.43	up	alpha-amylase 1 [Triticum aestivum] 6B
TraesCS6B02G364900	10.29	up	4.92	up	alpha-amylase 1 [Triticum aestivum] 6B
TraesCS6B02G349800	12.69	up	5.81	up	alpha-amylase 1 [Triticum aestivum] 6B
TraesCS6B02G364800	13.83	up	6.38	up	alpha-amylase 1 [Triticum aestivum] 6B
TraesCS7B02G286700	1.36	up	1.38	up	alpha-amylase 2 [Triticum aestivum] 7B
TraesCS5A02G238100	3.11	up	3.61	up	alpha-amylase 4 [Triticum aestivum] 5A
TraesCS5B02G236600	2.88	up	3.19	up	alpha-amylase 4 [Triticum aestivum] 5B
TraesCS7D02G168000	-1.08	down	-1.32	down	alpha-amylase/trypsin inhibitor CM1 7D
TraesCS7B02G072000	-1.48	down	-1.69	down	alpha-amylase/trypsin inhibitor CM2 7B
TraesCS1D02G254500	1.7	up	1.26	up	gibberellin receptor GID1-like 1D
TraesCS1B02G265900	1.35	up	1.68	up	GID1 protein [Triticum aestivum] 1B
TraesCS7B02G115300	4.46	up	3.59	up	gibberellin-regulated protein GASR7
TraesCS7A02G208100	5.26	up	4.34	up	gibberellin-regulated protein GASR7
TraesCS5D02G244900	6.7	up	5.47	up	ABA 8'-hydroxylase 5D
TraesCS5B02G236500	6.94	up	4.66	up	ABA 8'-hydroxylase 5B
TraesCS3D02G364900	-1.15	down	-1.22	down	bZip type transcription factor TaABI5 3D
TraesCS3B02G404400	-1.18	down	-1.51	down	bZip type transcription factor TaABI5 3B

Supplemental Table 12 The field environment data of Hefei, China (31° 58′ N, 117° 240′ E) during the 2017-2018 wheat cropping seasons.

Date (month/year)	Mean high temperature	Mean low temperature	average precipitation
10/2017	18 °C	12°C	97.5mm
11/2017	16 °C	7°C	10.8mm
12/2017	10 °C	0°C	13.6mm
1/2018	4 °C	-2°C	97.6mm
2/2018	9 °C	0°C	54.1mm
3/2018	17 °C	7°C	103.3mm
4/2018	23 °C	12°C	92.1mm
5/2018	26 °C	18°C	164.0mm

The temperature data source from website (https://www.tianqi.com/). The precipitation data source from website (http://www.stats.gov.cn/tjsj/ndsj/).
